# Verb-Mediated Prediction in Bilingual Toddlers

**DOI:** 10.3389/fpsyg.2021.719447

**Published:** 2021-11-11

**Authors:** Ane Theimann, Ekaterina Kuzmina, Pernille Hansen

**Affiliations:** ^1^Department of Linguistics and Scandinavian Studies, University of Oslo, Oslo, Norway; ^2^Center for Multilingualism in Society Across the Lifespan, University of Oslo, Oslo, Norway; ^3^Department of Humanities, Inland Norway University of Applied Sciences, Hamar, Norway

**Keywords:** semantic prediction, sentence processing, visual world paradigm (VWP), eye-tracking, bilinguals, toddlers, children

## Abstract

Prediction is an important mechanism for efficient language processing. It has been shown that as a part of sentence processing, both children and adults predict nouns based on semantically constraining verbs. Language proficiency is said to modulate prediction: the higher proficiency, the better the predictive skill. Children growing up acquiring two languages are often more proficient in one of them, and as such, investigation of the predictive ability in young bilingual children can shed light on the role of language proficiency. Furthermore, according to production-based models, the language production system drives the predictive ability. The present study investigates whether bilingual toddlers predict upcoming nouns based on verb meanings in both their languages, and whether this ability is associated with expressive vocabulary. Seventeen Norwegian-English bilingual toddlers (aged 2;5–3;3), dominant in Norwegian, participated in the study. Verb-mediated predictive ability was measured *via* a visual world paradigm (VWP) experiment, including sentences with semantically constraining and neutral verbs. Expressive vocabulary was measured by MacArthur-Bates CDI II. The results suggested that the toddler group predicted upcoming noun arguments in both their dominant and non-dominant languages, but were faster in their dominant language. This finding highlights the importance of language dominance for predictive processing. There was no significant relationship between predictive ability and expressive vocabulary in either language.

## Introduction

One of the reasons why auditory language processing is so efficient is linguistic prediction, which implies pre-activation of linguistic input before it has been uttered (Huettig, 2015; Karaca et al., 2021). A growing body of research has shown that both children (Borovsky et al., 2012; Mani and Huettig, 2012; Mani et al., 2016) and adults predict upcoming linguistic input during auditory language comprehension (Altmann and Kamide, 1999; Hintz et al., 2017; Ito et al., 2017). Karaca et al. (2021) suggest that language proficiency facilitates prediction, while Mani and Huettig (2012) argue that the predictive ability is connected to language production and, as such, to expressive vocabulary.

Children growing up acquiring two languages can possibly have a substantial variance in proficiency between these languages. In addition, they can have highly different expressive vocabulary sizes in the two languages (Conboy and Thal, 2006). Hence, investigation of language prediction in children who acquire more than one language could potentially shed more light on the factors contributing to its development. Studies investigating prediction in language comprehension in bilingual children are few (Brouwer et al., 2017; Lemmerth and Hopp, 2019; Meir et al., 2020), and to the best of our knowledge, no studies have focused on this ability in bilingual toddlers.

In their seminal work, Altmann and Kamide (1999) discovered that monolingual English-speaking adults looked toward a specific object faster when they were given a verbal cue to which object would be mentioned. In their visual world paradigm (VWP) experiment, the adult participants listened to audio stimuli consisting of sentences such as *The boy will eat/move the cake*, while they looked at visual stimuli depicting different objects where one was the target. For example, with respect to the aforementioned sentence: a boy, a cake, a ball, a toy train, and a toy car. While all of the objects depicted were *movable*, only the cake was *edible.* The researchers found that the participants’ gaze moved toward the cake faster upon hearing the semantically constraining verb *eat* than the more semantically neutral verb *move*. These findings were in favor of the hypothesis that adults predict nouns based on the semantic relationship between verbs and nouns; the adults in this study predicted upcoming nouns based on the semantic restrictions of verbs.

Previous studies also provide ample evidence for presence of predictive processing in monolingual children as young as 2 years old. Mani and Huettig (2012) employed the VWP to investigate whether monolingual German-speaking toddlers could use semantic cues represented by verb meanings to predict upcoming nouns. The toddlers listened to sentences such as *The boy eats/sees the big cake*, while looking at a screen with two pictures, where only one object was *edible*. The toddlers made predictive eye movements upon hearing semantically restrictive verbs (e.g., *eat*), but not when hearing non-restrictive verbs (e.g., *see*). Similarly, Borovsky et al. (2012) found that monolingual English-speaking children aged 3–10 years predict nouns based on verbs as well as sentential theme. The children were presented with four pictures (e.g., a treasure, a ship, a bone, and a cat), while hearing sentences such as *The pirate hides the treasure* or *The dog hides the bone*. In addition to semantic cues, there are other available cues to pre-activate upcoming linguistic input, such as prosodic, phonological, and morphosyntactic cues. An example of a morphosyntactic cue is grammatical gender, which can be used already by young children to predict upcoming nouns. Lew-Williams and Fernald (2007) showed that already by the age of 3, Spanish-speaking monolinguals identified target objects faster based on the gender-marked articles (el/la). The children heard sentences such as *Encuentra la pelota* ‘Find the_*FEM*_ ball’, and saw two pictures of objects that were either both feminine or of differing grammatical gender. The children found the object faster in the different-gender trials, suggesting that they used the gender-marked article as a cue.

The use of gender-marked articles to predict upcoming nouns has also been studied in adult bilinguals and L2 learners. The results are conflicting. Lew-Williams and Fernald (2010) used the same VWP study as with the 3-year-old described above, to investigate if adult English-speaking L2 learners of Spanish had the ability to predict based on the articles in Spanish. The adult L2-learners in this study did not predict based on gender-marked articles. In a study with the same design as Lew-Williams and Fernald (2010), but with more experienced L2 speakers of Spanish, Grüter et al. (2012) found that the L2 speakers used the gender marked article to predict familiar nouns. However, the L2 speakers were less efficient in their use of the predictive cue compared to native speakers of Spanish. Surprisingly, the L2 learners were better at using the gender marked article to predict novel nouns than they were with familiar nouns. In another study on adult English-speaking L2 learners of Spanish, Dussias et al. (2013) found that more experienced L2 learners predicted based on grammatical gender, whereas those with less experience did not.

Investigations on adult bilingual’s ability to predict have looked at not only morphosyntactic cues, but also semantic cues. For instance, Dijkgraaf et al. (2017) used the VWP to investigate late bilingual adults’ ability to predict upcoming nouns based on the semantic relationship between verbs and nouns. Dutch-English bilinguals (with dominance in Dutch) and a control group of English monolinguals heard sentences such as *Mary knits/loses a scarf*, while looking at a screen depicting four objects where all could be *lost*, but only one was *knittable*. Dijkgraaf et al. (2017) argue that it is important to test bilinguals’ predictive ability in both languages, due to individual differences connected to this ability, such as proficiency and vocabulary sizes. The researchers found that the bilinguals predicted based on semantic cues in both languages, but slower than the monolinguals (prediction effects reached significance 100 ms later in both languages). In a more recent study, Dijkgraaf et al. (2019) investigated Dutch-English bilinguals’ ability to predict upcoming semantic information in both their languages. The bilinguals saw four pictures, where three were distractor pictures and the fourth was either the target picture or a semantically related competitor. For instance, when the bilinguals heard the sentence *Her baby doesn*’*t like to drink from a bottle*, the target picture was a bottle, but in the semantically related trial it was a picture of a glass. The researchers found that the bilinguals predicted the semantics of target words in both conditions and in both languages. However, the prediction effect size was larger in the L1 than in the L2. Hopp (2015) investigated whether adult English L2 learners of German make predictions based on morphosyntactic cues (i.e., case marking) and verb semantics. The results showed that the bilinguals did not use morphosyntactic cues to predict, but they did use semantic cues.

Bilinguals are seldom completely balanced between their languages, neither in use nor in proficiency (Grosjean, 1989). Karaca et al. (2021) postulate that *language proficiency* modulates predictive processing: More proficient monolingual children and L2 learners are more likely to predict during sentence comprehension. Furthermore, based on previous studies on the predictive ability in monolingual children and adult L2 leaners, the researchers argue that language proficiency modulates the predictive ability in both L2 and L1. Similarly, Kaan (2014) argues that language users’ *lexical representations*, specifically the quality of these representations, affects the ability to predict. The researcher defines the quality of lexical representations as stability and accuracy of the language users’ knowledge of it—its form, meaning, and use. Thus, lexical representations of higher quality have fewer lexical competitors and will be activated and chosen faster and/or more accurately during language processing. Further, Kaan (2014) argues that through *exposure* to a specific language, one learns to associate certain linguistic elements with each other, and one stores the frequency of how often specific linguistic information occurs in the same context. Bilinguals are intriguing in this respect: with exposure divided between two languages, both linguistic representations and associations between them might be weaker, potentially affecting the ability to predict upcoming linguistic elements (Kaan, 2014). She further argues that although predictive processing in an L2 is similar to that in L1, it might be affected by less language exposure. Similarly, the weaker links hypothesis (Gollan et al., 2008) postulates that since bilinguals divide their time between two languages, they have weaker links between semantics and phonology in both languages compared to monolingual peers. These weaker links could result in a reduced ability to predict. From these assumptions, one would expect individual variation between bilinguals, depending on their exposure, proficiency, and use of each of their languages. Thus, increased language exposure and use and a higher proficiency could lead to more efficient predictive processing.

The studies described above investigated the predictive ability in adults speaking more than one language. To date, there are few studies devoted to predictive processing in bilingual children, especially of children younger than 3 years old. Lemmerth and Hopp (2019) tested whether bilingual Russian-German 8- and 9-year-old could predict upcoming nouns based on gender-marked articles (der/die/das) in German, and compared them to monolingual German children (also aged 8–9 years). The study included both simultaneous bilinguals, who acquired two languages from birth, and sequential bilinguals, who acquired or one from birth and another later on, The children heard sentences such as *Wo ist der/die/das gelbe [N]?* ‘Where is the_*MASC/FEM/NEUT*_ yellow [N]?’, while looking at pictures of four objects, of which only one had the grammatical gender mentioned in the sentence. The researchers found that the simultaneous bilingual children could use gender information to predict regardless of gender congruency, while the successive bilingual children would only predict when there was gender congruency between the two languages. Meir et al. (2020) investigated whether Russian-Hebrew bilingual children (4–8 years old) had the ability to predict upcoming nouns based on case-marking cues, and compared them to monolingual Russian children (aged 3–6 years) and Hebrew children (aged 4–8 years). The children looked at pictures, for example of a cabbage, a bunny and a fox. The researchers found that the bilingual children predicted based on case markers in Russian, as they looked at the agent (e.g., the fox) of the sentence upon hearing the accusative-marked NP (e.g., the bunny). However, they were slower than the monolingual Russian children. At the same time, the bilinguals used the case markers to predict also in Hebrew, whereas monolingual Hebrew children did not—as case-marking cues are assumed to be weighted lower than word order in Hebrew. Brouwer et al. (2017) tested the predictive ability based on semantic cues, employing the VWP, in Dutch monolingual and bilingual 4- and 5-year-old. The bilinguals spoke a variety of languages in addition to Dutch, but were only tested in Dutch. Of the bilinguals, 85% had learned Dutch before or around their first birthday, and their proficiency in Dutch was ranked as high. The children heard sentences such as *The boy eats/sees the big cake* while being presented with visual stimuli depicting two objects, where only one was edible. Brouwer et al. (2017) found that all the children (4- and 5-year-old monolinguals and bilinguals) predicted upcoming noun arguments based on the semantics of verbs. The researchers also found that the 4-year-old bilinguals predicted faster than their monolingual peers.

Although the number of studies on factors mediating predictive linguistic processing in bilingual children is relatively sparse, there are theories attempting to account for mediating factors of this ability for children and adults. According to production-based models, it is the production system that drives this ability (Pickering and Garrod, 2013; Pickering and Gambi, 2018). The foundational assumption of this theory is that the comprehension and the production systems are interwoven, allowing us to covertly imitate the speaker’s production and predict their next word (Pickering and Garrod, 2013). Huettig (2015) sees production as an underlying mechanism for the predictive ability, and argues that in predictive processing we use “fully specified production representations” (p. 125).

Several studies have indeed shown a link between production (expressive vocabulary) and prediction. For instance, in a study by Ito et al. (2017), for half of the trials in a VWP experiment, the researchers asked the participants to just listen to audio stimuli, and for the other half they asked them to listen and shadow (i.e., repeat the sentence back as fast as they could). The researchers found that predictive eye movements happened earlier during the shadow tasks than during the listen tasks. They concluded that the study supports the hypothesis that production facilitates prediction. The link between production and predictive processing has also been found in studies with monolingual toddlers. For instance, Mani and Huettig (2012) showed that monolingual toddlers with larger expressive vocabularies (i.e., from 225 words) showed predictive eye movements suggesting that they were able to employ semantic cues for predictive processing. At the same time, the toddlers with smaller expressive vocabularies (i.e., fewer than 225 words) did not show predictive eye movements. Similarly, Mani et al. (2016) found that monolingual toddlers with larger expressive vocabularies had significantly more looks to the target picture during the predictive window, compared to children with smaller expressive vocabularies.

More studies investigating the predictive ability in bilingual children could help shed light on the role of proficiency and exposure to the languages in question. Compared to their monolingual peers, simultaneous bilinguals typically have larger *total vocabularies* (i.e., total sum of words known from all languages), comparable *conceptual vocabularies* (i.e., concepts that they have a word for in either one or both languages), and smaller language-specific vocabularies (Pearson et al., 1993; De Houwer et al., 2014). It is well-established that early grammatical development depends on lexical development (Bates and Goodman, 2001; Devescovi et al., 2005), a connection that holds *within each language* for bilinguals (Conboy and Thal, 2006). At the same time, studies of cross-linguistic influence point toward cognitive permeability between languages for simultaneous bilinguals (Döpke, 2001; Hulk, 2001), meaning that the processing of input in one language can indeed influence the acquisition of the other, as long as there is structural overlap between them. The Unified Model (MacWhinney, 2008, 2012) postulates that words that appear together, for example, verbs and nouns that often occur together, map on to each other. Words acquired in a non-dominant language may benefit from mappings made in the dominant language. Furthermore, according to the Unified Model, there is extensive transfer of knowledge from the dominant to the non-dominant language. Thus, an intriguing question is whether bilingual children’s predictive ability relies on *within-language* vocabulary, as grammatical development in general, or the vocabulary in the strongest language, if predictive abilities in the non-dominant language comes as a result of cross-linguistic influence.

Simultaneously bilingual children are interesting for another reason: while we see great variability in the lexical development of monolingual children, there is reason to expect even more variability among bilinguals. They may be balanced between their languages or stronger in either, depending on a variety of factors, including the family language policy and the societal attitudes toward their languages. Hence, data from simultaneous bilinguals can potentially illuminate the relationship between prediction and expressive vocabularies. It is therefore important to look at both languages of the bilingual children. To date, there have been no studies investigating predictive ability based on semantic cues in both languages of bilingual children, and no studies at all investigating this ability in bilingual toddlers.

### The Current Study

In the current study, we investigate verb-mediated prediction in a group of Norwegian-English bilingual toddlers dominant in Norwegian, and its relationship with their expressive vocabularies in both languages. Norwegian and English are structurally similar languages, with SVO (i.e., Subject-Verb-Object) order, which makes it possible to investigate verb-mediated prediction within the same sentence structure across languages. The study considers the following two research questions:

(1) Do Norwegian-English bilingual toddlers use verb meanings to predict upcoming noun arguments in Norwegian and/or English? If they do, is there a difference in speed of predictive processing between the dominant (i.e., Norwegian) and non-dominant (i.e., English) language?
(2) Is the linguistic predictive ability associated with linguistic production skills, specifically expressive vocabulary?

Based on previous studies, we hypothesized that the group of Norwegian-English bilingual toddlers: firstly, would predict noun arguments based on semantically constraining verbs in their dominant language (i.e., Norwegian). Secondly, we hypothesized that the toddlers would either not predict in their non-dominant language (i.e., English) or predict significantly slower compared to their dominant language.

With regard to the second research question, we hypothesized that we would find a significant positive association between the predictive ability in one language and expressive vocabulary size in the same language. This hypothesis was based on Mani et al. (2016) reporting the predictive ability to correlate with expressive vocabulary, and the findings of Conboy and Thal, 2006 indicating that lexical development in one language primarily leads to better grammatical abilities in the same language.

In addition to the two main research questions, we had one exploratory goal. Given that bilingual children acquire words from two languages at the same time, we explored relationships between the total vocabulary (i.e., the total sum of words in both languages) and predictive ability in either language.

## Materials and Methods

### Participants

We recruited 18 simultaneous Norwegian-English bilingual toddlers for the study. We excluded one participant due to poor comprehension of the stimuli in the eye-tracking experiment, leaving us with 17 participants (6 females and 11 males) aged 2;5–3;3 (years;months) (*M* = 2;8, *SD* = 0.26). We recruited the toddlers *via* personal networks and posts on social media. To collect information on each toddler’s language background, their parents filled out an electronic questionnaire based on the Parent of Bilingual Children Questionnaire (PaBiQ) (COST Action IS0804, 2011; Norwegian version; Hansen and Simonsen, 2016). We used their responses to calculate the balance in the children’s language exposure, following Hansen et al. (2019). According to this calculation, all the toddlers were dominant in Norwegian. Corroborating this analysis, the parents of all the toddlers reported that their child felt most at home in Norwegian.

All the toddlers lived in or close to Oslo, and went to Norwegian-speaking day care. None of the toddlers used glasses, nor did any of them have hearing impairments or frequent ear infections. The toddlers’ expressive vocabularies were assessed by the parents filling out the electronic version of the parental report tool MacArthur-Bates Communicative Development Inventories Words and Sentences (i.e., MB-CDI II),^1^ developed for 16– to 36-month-old children in Norwegian (Kristoffersen and Simonsen, 2012), and for 16– to 30-month-old children in English (Fenson et al., 2007). In line with the reports on language dominance, all the toddlers had larger vocabularies in Norwegian (ranging from 281 to 664 words, *M* = 549.41, *SD* = 95.54) than in English (ranging from 20 to 565 words, *M* = *217.56*, *SD* = 156.97). The toddlers’ vocabulary scores, including information about age and gender, is given in Supplementary Table 1. Parents gave written informed consent before participation in the study. The toddlers received a small toy after completing each session. Prior to commencing data collection, the Norwegian Center for Research Data (NSD) had approved the study.

To ensure the reliability of the eye-tracking experiment, we piloted it on 20 Norwegian-English bilingual adults (8 females and 12 males) aged 22–58 years (*M* = 28.4, *SD* = 7.4). These participants gave written consent to participate in the current study, and had the chance to win a gift certificate.

### Materials

#### Eye-Tracking Experiment

**FIGURE 1:**
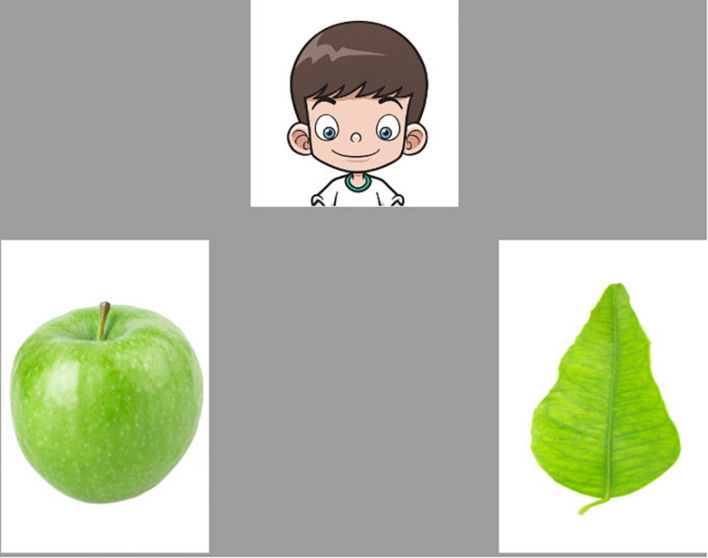
Example of the visual stimuli for the auditory stimulus *This is a boy. The boy eats the green apple*.

The eye-tracking experiment employed the VWP and consisted of 56 stimulus sets, divided into four lists, two in each language, with14 stimuli sets in each list. Each stimuli set included one audio stimulus (i.e., either a semantically constraining or a neutral sentence—e.g., *The boy* ***eats/takes*** *the green apple*) and one visual stimulus, with three corresponding pictures: a picture of a boy/girl displayed in the top middle part of the screen, which functioned as a fixation picture, and the picture pairs (target and distractor) displayed lower on the right and left sides of the screen (see Figure 1). Including a fixation picture of the sentential subject made the participants look toward that picture at the start of the sentence. This minimized the possibility that the participants looked toward the target picture too early or by chance. We placed the fixation picture at the top middle at the screen because it is more natural to look at the top of the screen first. In the fixation pictures, both the boy and the girl looked straight down, so that the participants would not be biased toward any of the pictures. The picture pairs were bigger (9.7 cm°×°6.5 cm) than the fixation picture (5.5 cm°×°5.5 cm). We edited the pictures with Gimp, version 2.8 (The GIMP Team, 2018), so that all the pictures had a white background and no shadows. We found most of the pictures in the picture database Colourbox.com (2018), and the rest via searches on the internet.

In every picture pair, the pictures had similar size, color, and shape, and both were either photos or vectors. If the target picture was animate, the distractor picture was also animate. The object in the distractor picture was semantically and associatively unrelated to the object in the target picture and to the constraining verb. The reason behind this was that previous VWP studies have shown that participants tend to look more toward visual objects that are semantically related to the spoken word, than to visual objects that are unrelated (Huettig et al., 2006). The reason for only including two pictures (target and distractor) in addition to the fixation picture was to prevent toddlers from becoming overwhelmed by the visual stimuli. The position of the target picture appearing on the right or on the left side of the screen (see Figure 1) was counterbalanced. The position of the target picture was also counterbalanced across conditions.

For each language, 14 sentence pairs were created (all sentences can be found in Supplementary Table 2). A sentence pair consisted of one sentence with a semantically constraining verb (e.g., *eats*) and one sentence with a neutral verb (e.g., *takes*). Within one language, each verb was used only once in the experiment. The same semantically constraining verbs were used in both languages. However, the noun arguments following the verbs differed between the languages. For example, the corresponding sentence pair for *The boy* ***eats/takes*** *the orange carrot* in English was *Gutten* ***spiser/tar*** *det grønne eplet* ‘The boy **eats/takes** the green apple’ in Norwegian. Using different noun arguments ensured that participants saw each picture pair only once during the entire experiment consisting of two sessions. For example, for the sentence pairs described above, the target pictures were a green apple or an orange carrot in the Norwegian and English tasks respectively. We designed the sentences with neutral verbs so that the verbs would plausibly fit with both pictures; for instance, if the verb was *pick up*, it was possible to pick up both objects in the pictures. In some cases, we had to include a preposition after the verb for the stimulus sentences to ensure grammatical correctness (e.g., *The girl sits* ***on*** *the cold bench*), or to make verbs semantically constraining (e.g., *The girl draws* ***with*** *the blue crayon*).

A short context sentence preceded each sentence, namely *Her er det en jente/gutt* ‘Here there is a girl/boy’ in Norwegian and *This is a girl/boy* in English. The context was neutral, so the toddlers were not primed to look at the target or distractor pictures. From experience, toddlers are easily distracted when they hear names of family members or friends, we therefore decided that all the sentences should start with *The boy* or *The girl.*

The experimental material consisted of four lists: two in Norwegian and two in English, created to balance the visual and auditory stimuli across the participants, while also avoiding repetition. The two lists from each language were evenly distributed among the toddlers. In other words, one list from each language was used for half of the toddlers. Equally many sentences in each list began with *The boy* and *The girl*, half of which had semantically constraining and neutral verbs, respectively. Each child saw each picture pair only once. Trials were fully randomized on a by-participant basis by the experimental software.

A Norwegian-English bilingual female speaker recorded the auditory stimuli in a quiet environment with a Zoom Q2n, with 48,000 Hz. We asked her to record the auditory stimuli because of her clear pronunciation of Norwegian and English, and because she had partly been growing up in an English-speaking society and partly in a Norwegian-speaking society. To edit the auditory stimuli we used Audacity, version 2.2.2 (Audacity Team, 2021). We edited the length of a short pause (*M* = 812.47 ms; *SD* = 210.74) after the context sentence, to make sure the verb onset was at exactly 3,500 ms into the trial. Similarly, we edited the length of another short pause (*M* = 564.23 ms; *SD* = 143.23) after the verb, to make sure the noun onset was at 5,300 ms. This gave the toddlers a time window of 1,800 ms to predict the upcoming noun (e.g., This is a boy. [pause] The boy eats [pause] the green apple). No linguistic cues appeared during the predictive window that could bias the toddlers to look toward either of the pictures.

All the adult pilot participants completed the Norwegian version first, and the English version about 2 weeks later. After each session, we asked the participants how sentences sounded to them and whether the visual stimuli fitted the sentences. Six adults noted that the audio stimuli sounded child-directed. All agreed that the sentences sounded natural, that the pictures matched the task, and that the two pictures shown at the same time were equally salient.

### Procedure

The toddlers performed the eye-tracking task either in the Socio-Cognitive laboratory at the University of Oslo, or at the toddler’s daycare center. The toddlers performed the eye-tracking task twice—first in Norwegian, and 1–2 weeks later in English. At the beginning of each session, we introduced the toddlers to a stuffed animal, and told them that the stuffed animal could only speak the language of that session. Toddlers sat on their parent’s lap, facing a monitor. The eye-tracker was located underneath this monitor. Eye movements were recorded from the right eye with an SMI RED25mobile Eye Tracker, with a sample rate of 250 Hz. Auditory stimuli were presented through a speaker, connected to the monitor. We controlled and monitored the experiment by a laptop computer. The experimenter instructed the parents to sit still and not say anything during the experiment, so as to not guide the toddlers to look at any of the pictures.

We used an image of a bee, shrinking in on itself, to calibrate the toddlers’ eye movements. To make the calibration more playful, the experimenter told the toddlers that the bee would fly for them if they looked at it. After a successful calibration, the experimenter told the toddlers that they would hear some stories about a boy and a girl, and that they should look at the pictures on the monitor in front of them. After the calibration, two practice trials followed. After each trial, we asked the toddlers to point to the pictures that matched the sentence they had just heard. Previous studies have shown that young children pay more attention performing eye-tracking tasks when they are asked to point to the correct picture after each trial (Trueswell, 2008). During the practice trials, if the toddlers did not point to the target picture, we repeated the practice trials until they did. In each trial, the three pictures appeared and stayed on the screen for 1,000 ms before the auditory stimuli started. Once the participant had pointed to a picture, the next trial was started by the experimenter. If a toddler pointed to the distractor picture, we interpreted it as incorrect sentence understanding. After half of the trials, a new calibration started, and the toddlers that needed it had a break before the second calibration. Each session lasted about 15 min. Within a week of each test session, the parents filled out the CDI form for the language tested that day.

### Data Analysis

For the analysis, we only used data from trials where the toddlers understood the sentences correctly. As previously mentioned, if a toddler pointed to the distractor picture at the end of a sentence, we interpreted it as incorrect sentence understanding. Therefore, 9% of the trials were excluded (43 out of 476). All of the adults understood all of the sentences, so we kept all trials in the pilot study. We used both fixations and saccades in the analysis because young children have less stable patterns of eye-movements and tend to saccade within areas of cognitive fixation (Aring et al., 2007). Conventionally, fixations are defined as time periods when eyes fixate on a specific area and stay relatively stationary—from tens of milliseconds to several seconds, while saccades are defined as rapid eye movements between any two fixations (Holmqvist et al., 2011). In the remainder of this paper, *fixations* will refer to both saccades and fixations used in the analysis.

We did not perform statistical analysis on the data from the adult group and relied on visual investigation of the fixation curves only, since the pilot study only served as a proof of concept for the chosen experimental design. For the analysis of the data from the toddler group, we used the divergence point analysis reviewed in great detail and with remarkable clarity by Stone et al. (2020). We encourage our readers to acquaint themselves with the before mentioned paper. The analysis script in the current study is adapted from the tutorial provided in Supplementary Material by Stone et al. (2020).

The main goal of the divergence point analysis is to allow researchers studying online unfolding of language comprehension to estimate specific timepoints of effect onsets. Once estimated, the effect onset timepoints can be directly compared between experimental groups and/or conditions in order to conclude in which experimental group and/or condition the effect onset manifests earlier. Several methods can be used to perform the divergence point analysis and these methods have their specific advantages and disadvantages (Stone et al., 2020). In the current study, we used Generalized Logistic Mixed-effect Models (GLMM; Barr, 2008) and our effect of interest was the onset of verb-mediated predictive processing. We operationalized its onset as the timepoint that (1) is located between the verb and noun onsets in the constraining condition and (2) corresponds to a significant increase in target-fixations compared to distractor-fixations.

To analyze the data, we first defined the critical predictive window as the time period between the verb onset + 300 ms and the noun argument onset + 300 ms. Given that, firstly, adults use at least 200 ms to launch a saccade (Altmann and Kamide, 1999; Salverda et al., 2014) and, secondly, that children are generally slower than adults at launching saccades (Bucci and Seassau, 2012; Lemoine-Lardennois et al., 2016), 300 ms were added to the verb onset as well as to the noun onset. We also added a buffer of 1,700 ms after the noun onset + 300 ms to detect a divergence point in the neutral condition where we did not expect any predictive processing.

Secondly, we used the GLMM. We fitted a generalized logistic mixed-effect model to data from each 20 ms time slot of this critical predictive window to compare binomial distributions of target and distractor fixations in each time slot. The time slot where a significant difference between the number of target- and distractor-fixations was observed for the first time was defined as the divergence point, specifically the onset of the verb-mediated processing. This method requires to run multiple statistical tests, which is associated with an increased risk of making a Type I error, that is, detecting an effect when it is in fact absent in reality. Therefore, to adjust for multiple comparisons, we used the false discovery rate (FDR) control correction (Benjamini and Hochberg, 1995) when fitting the model. We preferred the FDR correction to the Bonferroni correction, because the latter is associated with reduced statistical power and consequently increased probability to miss an effect that in fact exists in reality (i.e., a Type II error). The FDR correction makes *p*-values from each significance tests larger based on a specific algorithm. This results in a lower number of false positives passing the initially chosen alpha-level.

Following Mani and Huettig (2012), the ability to predict upcoming linguistic information (i.e., predictive ability) was operationalized as the difference in the number of target-fixations between the semantically constraining and neutral conditions within the critical predictive window. To measure the strength of the relationship between CDI vocabulary scores and predictive ability in each language separately, Spearman correlation coefficients were used. The same method was used to explore whether there is a relationship between the overall productive vocabulary and predictive ability. R (R Core Team, 2021) and RStudio (Rstudio Team, 2021) were used for the data analysis. The R script, detailed report of the analysis, and data can be found in the Supplementary Material.

## Results

### Results of the Pilot Study With the Adult Group

**FIGURE 2:**
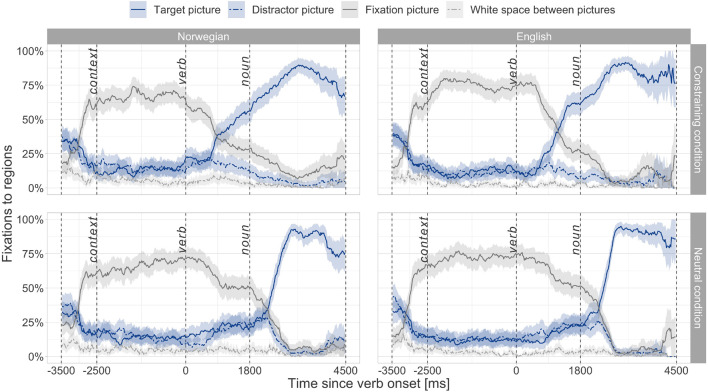
Fixation curves made of mean fixation percentages with 95% confidence intervals calculated per 20 ms time bins for the four areas of interest in the adult group. “*Context*” refers to the time point when a context sentence starts, “*verb*”—when a verb starts, and “*noun*”—when a noun argument starts.

The main goal of the pilot study was to ensure that the proposed experimental design captures verb-mediated prediction. As can be seen from Figure 2, the adult group showed predictive processing in both Norwegian and English: Percentages of target-fixations clearly increased (1) within the critical predictive window in the constraining condition and (2) after the noun onset in the neutral condition. Based on these results from the visual inspection of the fixation curves, we concluded that the proposed experimental design is suitable for studying verb-mediated predictive processing.

### Results of the Main Study With the Toddler Group

**FIGURE 3:**
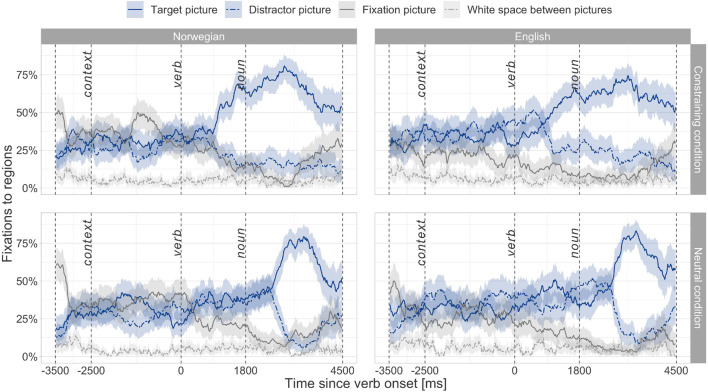
Fixation curves representing mean fixation percentages with 95% confidence intervals calculated per 20-ms time bin for the four areas of interest in the toddler group. “*Context*” refers to the time point when a context sentence starts, “*verb*”—when a verb starts, and “*noun*”—when a noun argument starts.

Figure 3 summarizes performance of the toddler group and displays a noticeable increase in target fixations happening within the critical predictive window in the constraining conditions well as after the noun onset in the neutral condition in both Norwegian and English. Thus, this descriptive plot already suggests that the toddler group predicted upcoming nouns based on verb meanings in both their dominant and non-dominant language. To estimate exactly when this verb-mediated predictive processing started, a divergence point analysis was performed.

**FIGURE 4:**
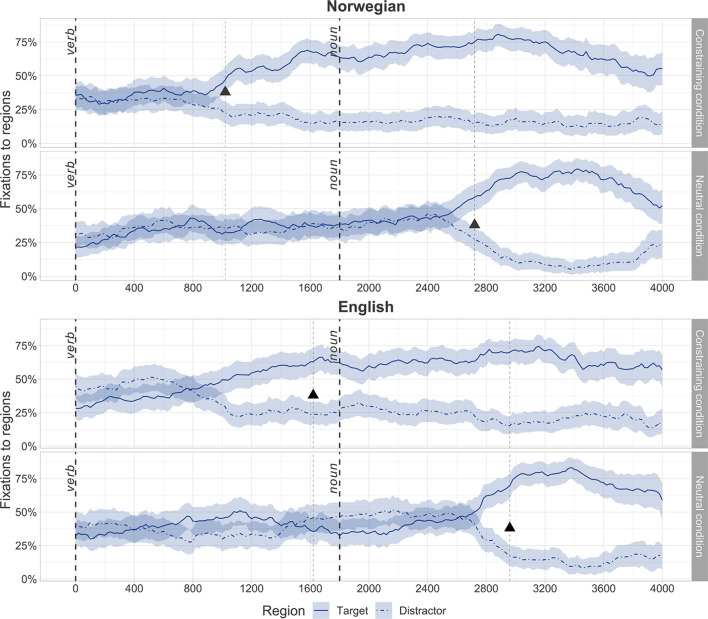
Fixation curves for the critical predictive window. Triangles depict the divergence point estimate calculated with the generalized logistic mixed-effect models (GLMM) using the false discovery rate (FDR) correction.

Figure 4 displays performance of the toddler group within the critical predictive window. The triangle point depicts the divergence point estimate calculated with the FDR correction. Table 1 summarizes the divergence point estimates for different conditions and languages in the toddler group.

**TABLE 1:** Summary of the divergence point estimates.

Condition	Norwegian			English			Difference between the divergence point estimates, ms
	Time after verb onset, ms	*z*-score	*p*-value	Time after verb onset, ms	*z*-score	*p*-value	
Constraining	1,020	3.00	<0.05	1,620	2.95	<0.05	600
Neutral	2,720	3.28	<0.05	2,960	3.49	<0.05	240

For Norwegian, the divergence point estimate was before the noun onset (i.e., 1,800 ms after the verb onset) in the constraining condition: 1,020 ms after the verb onset. As expected, in the neutral condition the divergence point estimate was after the noun onset pointing to the absence of predictive processing. These results suggest that the toddler group used verb meanings to predict upcoming noun arguments in their dominant language, Norwegian.

For English, the divergence point estimate was within the critical predictive window in the constraining condition: 1,620 ms after the verb onset. Similar to the Norwegian results, the FDR divergence point estimate in the neutral condition was outside of the critical predictive window pointing to the absence of predictive processing. These results provide evidence for verb-mediated predictive processing in the toddler group in their non-dominant language, English.

The toddler group was generally faster in both constraining and neutral conditions in their dominant language, Norwegian, compared to English. Specifically, in the neutral condition capturing verb-based integration of noun arguments, the toddlers were 240 ms faster in Norwegian. For the constraining condition this difference was 600 ms.

**FIGURE 5:**
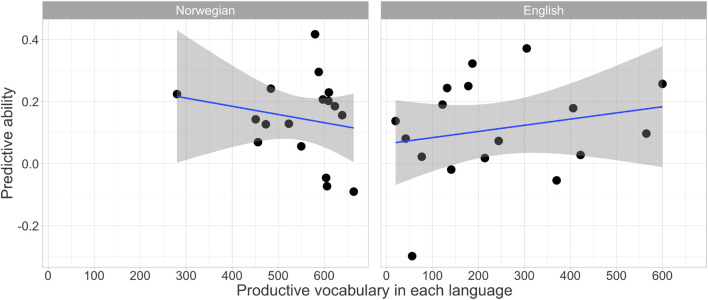
Correlations between the predictive ability and expressive vocabulary.

The predictive ability was calculated as the difference in the number of target-fixations between the semantically constraining and neutral conditions within the critical predictive window. There were no significant correlations between predictive ability and productive vocabulary in either Norwegian, *r* = –0.14, *p* = 0.59, or English, *r* = 0.19, *p* = 0.47. Figure 5 shows relationships between productive vocabulary and predictive ability in both languages.

**FIGURE 6:**
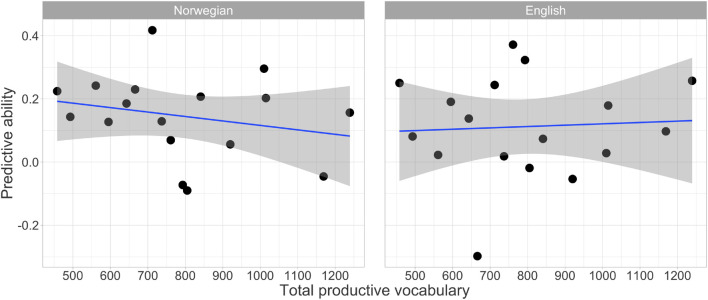
Correlations between the predictive ability and total expressive vocabulary.

There was also no significant relationship between the total productive vocabulary and predictive ability in either Norwegian, *r* = –0.26, *p* = 0.30, or English, *r* = 0.002, *p* = 0.996. Figure 6 shows relationships between total productive vocabulary and predictive ability in both languages.

## Discussion

The present study is the first to investigate the semantic predictive ability in bilingual toddlers in both their languages. We set out to answer two research questions. The first question was whether bilingual toddlers predict upcoming nouns based on verb meanings in both their dominant and non-dominant languages—namely Norwegian and English, respectively—and, if they do so, whether there is a difference in speed of predictive processing between the two languages. The second question was whether toddlers’ production skills, specifically expressive vocabulary, mediates this predictive ability. Linguistic predictive processing was investigated by means of an eye-tracking experiment employing the VWP. The expressive vocabulary sizes were assessed with the Norwegian and English MacArthur-Bates Communicative Development Inventories Words and Sentences (i.e., MB-CDI II). Below, we will discuss our findings in relation to these questions.

Concerning our first research question, we hypothesized, firstly, that the toddler group would predict noun arguments based on semantically constraining verbs in their dominant language (i.e., Norwegian). Secondly, we hypothesized that they would either not predict in their non-dominant language (i.e., English) or predict significantly slower compared to their dominant language.

The results from the current study support the first part of this hypothesis: As expected, in Norwegian the divergence point estimate was within the critical predictive window (i.e., after the verb- and before the noun-onset) in the constraining condition and outside of it in the neutral condition. As previously mentioned, the divergence point was defined as the first time slot with a significant difference between the number of target- and distractor-fixations. These results provide clear evidence for the presence of verb-mediated predictive processing of noun arguments in toddlers in their dominant language. These findings are in line with findings from previous research. In particular, previous studies have shown that monolingual toddlers at the age of 2 years (Mani and Huettig, 2012; Mani et al., 2016) as well as monolingual children aged 3–10 years (Borovsky et al., 2012) predict upcoming lexical information during sentence comprehension. Additionally, Brouwer et al. (2017) found that bilingual children aged 4 years old were able to predict in their majority language using semantic cues similarly to their monolingual peers.

The second part of our hypothesis connected to the first research question was also supported by the current findings: in English, the divergence point estimate was within the critical predictive window in the constraining condition and outside of it in the neutral condition. Thus, we concluded that the current study provides evidence for verb-mediated predictive processing in toddlers in a non-dominant language.

With regards to the speed of processing, the toddler group was generally faster in their dominant language, Norwegian, compared to their non-dominant language, English. In the constraining condition, which taps into predictive processing, the processing advantage for the domain language was 600 ms.

In English, the divergence point estimate were later in both neutral and constraining conditions in English compared to Norwegian (see Table 1). To the best of our knowledge, there are no existing studies on semantic prediction in bilingual toddlers in both their languages. However, the current results are in line with experimental evidence from studies with adults, finding that adult bilinguals predict slower in their L2 compared to monolinguals. For instance, Dijkgraaf et al. (2019) compared Dutch-English bilinguals’ L1 to their L2 and found that they predicted based on semantic cues slower in L2 than in L1. Chun and Kaan (2019) showed that adult Chinese L2 learners of English predicted 180 ms slower than native English speakers when listening to English. Dijkgraaf et al. (2017) investigated semantic verb-mediated prediction and identified that adult Dutch-English bilinguals predicted 100 ms slower in both of their languages compared to native English speakers.

The current study is the first to test semantic prediction skills in bilingual 2;5- to 3;3-year-old toddlers in both languages. By testing both languages with almost identical stimuli in the same individuals, it is possible to investigate the differences between the prediction abilities in two languages within individuals. This eliminates between-group differences (e.g., speed differences in eye movements, cultural and linguistic backgrounds, as well as individual cognitive differences), which is difficult to avoid when comparing bilinguals’ non-dominant language to native speakers (Dijkgraaf et al., 2019).

In the present study, the toddlers had less exposure to English than to Norwegian, potentially resulting in weaker mental representations in this language, and in turn slower prediction. These findings are in line with the theoretical accounts of Gollan et al. (2008), Kaan (2014), and Karaca et al. (2021), and indicate that semantic prediction is less efficient in the non-dominant language. Following the weaker links hypothesis (Gollan et al., 2008), the less efficient prediction could be due to weaker links between verb meanings and their arguments in the less practiced language. The current findings are also in line with the Unified Model (MacWhinney, 2008, 2012). Findings from studies of simultaneous bilingual language acquisition indicate that knowledge from one language can indeed influence the acquisition of another, as long as there is structural overlap. Since Norwegian and English are structurally similar languages, the toddlers who participated in our study could be transferring their ability to predict from the dominant to the non-dominant language. In fact, cross-linguistic influence in semantic (or conceptual) knowledge could be what allows them to predict in their non-dominant language at all. When the toddlers have understood that certain verbs are followed by certain noun arguments (e.g., eat and something edible) in their strongest language, they transfer this knowledge to the weakest language, and thus predict in this language as well. Thus, if a toddler knew the meaning of the verb *eat* in English, they could use their mental representations from Norwegian, their dominant language, to predict edible objects, even if they did not know the English names for the edible objects depicted on the screen.

Other studies of prediction with bilingual children also report findings that suggest transfer of knowledge from the dominant to the non-dominant language. Lemmerth and Hopp (2019) showed that Russian-German successive bilingual 8- and 9-year-old would only predict nouns based on grammatical gender in cases where there was gender congruency between Russian and German. The researchers argue that all nouns in both languages with the gender they heard get activated, so the nouns with gender congruency across languages benefited from the activation and eased prediction. At the same time, the nouns with incongruent gender suffered from competition effects. In a study with Russian-Hebrew bilinguals aged 4–8 years, the bilinguals were slower to predict based on case markers compared to their monolingual Russian-speaking peers (Meir et al., 2020). However, in contrast to Hebrew monolinguals, only the bilinguals used the case markers to predict in Hebrew. This indicates that the bilinguals transferred their case marker knowledge from Russian, where case markers are commonly used to predict, to Hebrew, where case markers are used less as a cue to predict.

To summarize, the results we obtained to answer our first research question, suggest that bilingual toddlers at the age of 2;6 are able to use verb meanings to predict upcoming noun arguments in both of their languages. However, they are faster at predicting in the language reported as their dominant one, where they have the largest vocabulary, according to parental reports.

The second research question was whether there was an association between predictive ability and production skills represented by expressive vocabulary. We hypothesized that there would be a positive correlation between the predictive ability and expressive vocabulary sizes; the toddlers with higher productive vocabulary sizes would predict faster. Previous studies on grammatical and lexical development in bilingual children indicate that lexical development in one language leads to better grammar in the same language only. Therefore, we expected a positive correlation between the predictive ability in one language and the expressive vocabulary in the same language.

The results did not support this hypothesis. There were no significant correlations between predictive ability and expressive vocabulary size within either of the two languages. This is in line with findings from Dijkgraaf et al.’s (2017) study, where the researchers did not find a significant relationship between predictive ability and production in the respective languages in a group of adult bilinguals. The researchers assumed that the method they used to measure bilinguals’ vocabularies (i.e., LexTALE) was not sensitive enough to reflect variation in production skills in the group of adults, and that, in turn, this could have explained the non-significant results they obtained. Previous studies have provided evidence for the production-based models, suggesting that one of the possible mechanisms for language prediction during sentence comprehension is language production skills (e.g., Mani and Huettig, 2012; Mani et al., 2016; Martin et al., 2018). For instance, Mani and Huettig (2012) found a positive correlation between monolingual 2-year-olds’ predictive ability and their productive skills. The toddlers with expressive vocabulary sizes from 225 words according to the MB-CDI had better predictive ability compared to the toddlers with smaller expressive vocabularies. Mani and Huettig argue that production skill is one of the underlying mechanisms, and that toddlers with larger expressive vocabularies will have stronger links between constraining verbs and their arguments, which in turn facilitates predictive processing. However, while the toddlers in the present study predicted, there was no link between the predictive ability and the same language vocabulary. We used the same method to measure expressive vocabulary as Mani and Huettig (2012). The obvious difference between the two studies is that their study concerned monolingual toddlers while and the present study concerned bilingual toddlers. As mentioned in the introduction, there are different ways to look at bilinguals’ vocabularies, which we will discuss below. The findings in the present study do not provide evidence in support of the theoretical account where productive vocabulary is seen as an underlying mechanism of the predictive ability.

In addition to the two research questions, we had an exploratory goal: to investigate whether there was a correlation between the predictive ability in each of the languages and the total expressive vocabulary. There was no correlation between the predictive ability in either of the languages and the total expressive vocabulary. It is possible that this result is an artifact of the measure used. As mentioned in the introduction, bilingual children’s lexical development can be assessed by first assessing their vocabulary in each language separately, and then use those data to calculate either a conceptual vocabulary or a total vocabulary (Pearson et al., 1993; De Houwer et al., 2014). Both of these measures have their advantages. This study calculated the total vocabulary, that is, the sum of words produced in Norwegian and English. The measure requires no qualitative judgment from the researcher, but may give inflated numbers, particularly for children who know many cognates, that is, words from different languages overlapping in form and meaning. Hence, a child scores 2 points for producing both the Norwegian word *banan* and its English equivalent *banana*, and 1 point for producing the Norwegian word only. The measure of conceptual vocabulary, on the other hand, is meant to reflect the number of concepts the child has a word for, regardless of which language the concept is in. Here, a child would score only 1 point whether they produced both the Norwegian *banan* and the English *banana* or only one of these words. This measure is more conservative, not inflated by the existence of cognates, and it may yield a more valid picture of bilingual children’s lexical knowledge. However, the calculation requires the researcher to map conceptual equivalents among the words that the children produce in their two languages. This task is not trivial, as complete conceptual equivalents across languages are rare (Pavlenko, 2009; de Groot, 2013). Some words may seem to have an absolute conceptual equivalent, but a complete overlap for all the uses in a range of situations and contexts of the word is hard to find. Thus, establishing a child’s conceptual vocabulary size is not straightforward. However, it is nevertheless possible that it is a more relevant measure for studies of verb-mediated prediction.

The current study is not without limitations. The first limitation we wish to address regards participant groups. Future studies could include a group of English-Norwegian simultaneous bilingual toddlers, to see if they would have the opposite results from the toddlers in this study: if they would show faster predictive abilities in English. Including a group of simultaneous bilingual toddlers with larger vocabularies in English than in Norwegian could help answering the question if proficiency is key to prediction. Future studies with older and more proficient bilingual children could shed light on whether the predictive ability in their non-dominant language would increase with increased proficiency in this language. In addition, more research on prediction in bilingual children at different ages, and with different proficiency levels between their languages would increase our knowledge on the role of proficiency for predictive ability. Another limitation of the current study is that we did not measure the toddlers’ receptive vocabulary or other receptive language processing skills. The current study focused on the relationship between the predictive ability and production, however there are also models that link prediction to comprehension (Chang et al., 2006; Gambi and Pickering, 2013; Dell and Chang, 2014; Ness and Meltzer-Asscher, 2021). Future studies should investigate a relationship between the predictive ability and both production and comprehension. For both modalities, the measure of conceptual vocabulary may be more suitable than the total vocabulary measure used here. Concerning our statistical analyses, the main drawback of GLMM is its inability to measure variability of the divergence points. As such it is not suited for statistical comparison of these points between conditions and/or groups. In a recent paper, Stone et al. (2020) suggest using bootstrapping to enable estimation of uncertainty around the divergence points. While it is uncertain how well-suited a limited dataset with high variability such as ours is for bootstrapping, we see this as a promising direction for the field.

## Conclusion

To conclude, findings from the current study suggest that bilingual toddlers predict upcoming nouns based on the semantic restrictions of verbs in both their dominant and non-dominant languages. However, they are faster in their dominant language. These findings are in line with the weaker links hypothesis: less exposure and lower proficiency in the non-dominant language lead to weaker mental representations and associations, which, in turn, result in slower linguistic predictive processing. Due to young age and limited experience with the non-dominant language, a toddler’s ability to predict may still be developing in this language. Despite their lower proficiency in the non-dominant language the bilingual toddlers in our study still predicted in this language. Following the Unified Model, the prediction in the non-dominant language could be explained by a transfer of this ability from the dominant to the non-dominant language. Findings from previous studies concerning the possible association between the predictive ability and production are conflicting. The results from the current study do not lend support to the theoretical account where productive vocabulary is seen as an underlying mechanism of the predictive ability: there was no relationship between the predictive ability and the expressive vocabularies in either language or with total vocabulary. Based on the conflicting findings on the expressive vocabulary’s role on predictive ability, more research is needed to investigate this relationship further.

## Data Availability Statement

The original contributions presented in the study are included in the article/Supplementary Material, further inquiries can be directed to the corresponding author.

## Ethics Statement

The study was reviewed and approved by Norsk senter for forskningsdata (NSD). Written informed consent to participate in this study was provided by the participants, and in case of the toddlers, by their legal guardian/next of kin.

## Author Contributions

AT initiated and led the present project, from design *via* data collection to the writing process, provided the idea to investigate prediction in bilingual children, designed and revised the experiment, led the recruitment of participants, collected the data, and drafted the introduction, methods, and discussion sections. EK provided the idea to study verb-mediated prediction, performed the statistical analysis, and wrote and revised the parts on analysis and results. EK and PH contributed with suggestions on methodology. PH wrote parts of the introduction and discussion. All authors provided critical feedback and helped shape the study and the manuscript.

## Conflict of Interest

The authors declare that the research was conducted in the absence of any commercial or financial relationships that could be construed as a potential conflict of interest.

## Publisher’s Note

All claims expressed in this article are solely those of the authors and do not necessarily represent those of their affiliated organizations, or those of the publisher, the editors and the reviewers. Any product that may be evaluated in this article, or claim that may be made by its manufacturer, is not guaranteed or endorsed by the publisher.

## Footnotes

1 Since only MB-CDI II was used in this study, for the remainder of this paper *CDI* will be used to refer to *MB-CDI II*.

## References

[B1] Altmann G. T. Kamide Y. (1999). Incremental interpretation at verbs: restricting the domain of subsequent reference. *Cognition* 73 247–264. 10.1016/s0010-0277(99)00059-1 10585516

[B2] Aring E. Grönlund M. A. Hellström A. Ygge J. (2007). Visual fixation development in children. *Graefe’s Arch. Clin. Exper. Ophthalmol.* 245 1659–1665. 17453232 10.1007/s00417-007-0585-6

[B3] Audacity Team (2021). Audacity(R): Free Audio Editor and Recorder [Computer Software]. Version 2.2.2. Available online at: https://audacityteam.org/ (accessed August 31, 2018).

[B4] Barr D. J. (2008). Analyzing ‘visual world’ eyetracking data using multilevel logistic regression. *J. Mem. Lang.* 59 457–474. 10.1016/j.jml.2007.09.002

[B5] Bates E. Goodman J. C. (2001). “On the inseparability of grammar and the lexicon: evidence from acquisition,” in *Language Development: The Essential Readings*, eds Tomasello M. Bates E. (Hoboken, NJ: Blackwell Publishing), 134–162.

[B6] Benjamini Y. Hochberg Y. (1995). Controlling the false discovery rate: a practical and powerful approach to multiple testing. *J. R. Statist. Soc. Ser. B* 57 289–300.

[B7] Borovsky A. Elman J. L. Fernald A. (2012). Knowing a lot for one’s age: vocabulary skill and not age is associated with anticipatory incremental sentence interpretation in children and adults. *J. Exper. Child Psychol.* 112 417–436. 10.1016/j.jecp.2012.01.005 22632758 PMC3374638

[B8] Brouwer S. Özkan D. Küntay A. C. (2017). “Semantic prediction in monolingual and bilingual children,” in *Cross-Linguistic Influence in Bilingualism*, eds Blom E. Cornips L. Schaeffer J. (Amsterdam: John Benjamins), 49–74.

[B9] Bucci M. P. Seassau M. (2012). Saccadic eye movements in children: a developmental study. *Exper. Brain Res.* 222 21–30. 10.1007/s00221-012-3192-7 22836522

[B10] Chang F. Dell G. S. Bock K. (2006). Becoming syntactic. *Psychol. Revi.* 113:234. 10.1037/0033-295x.113.2.234 16637761

[B11] Chun E. Kaan E. (2019). L2 Prediction during complex sentence processing. *J. Cult. Cogn. Sci.* 3 203–216. 10.1007/s41809-019-00038-0

[B12] Colourbox.com (2018). Available online at: https://www.colourbox.com (accessed August 15, 2018).

[B13] Conboy B. T. Thal D. J. (2006). Ties between the lexicon and grammar: Cross-sectional and longitudinal studies of bilingual toddlers. *Child Dev.* 77 712–735. 10.1111/j.1467-8624.2006.00899.x 16686797

[B14] COST Action IS0804 (2011). *Parents of Bilingual Children Questionnaire(PaBiQ). A Part of the LITMUS Battery (COST IS0804).*

[B15] de Groot A. M. B. (2013). “Bilingual memory: a short introduction,” in *The Psycholinguistics of Bilingualism*, eds Grosjean F. Li P. (Chichester: Wiley Blackwell), 171–191. 10.1002/brb3.2121

[B16] De Houwer A. Bornstein M. H. Putnick D. L. (2014). A bilingual–monolingual comparison of young children’s vocabulary size: evidence from comprehension and production. *Appl. Psycholinguist.* 35:1189. 10.1017/S0142716412000744 29527076 PMC5842817

[B17] Dell G. S. Chang F. (2014). The P-chain: relating sentence production and its disorders to comprehension and acquisition. *Philos. Trans. R. Soc. B Biol. Sci.* 369:20120394. 10.1098/rstb.2012.0394 24324238 PMC3866424

[B18] Devescovi A. Caselli M. C. Marchione D. Pasqualetti P. Reilly J. Bates E. (2005). A crosslinguistic study of the relationship between grammar and lexical development. *J. Child Lang.* 32:759. 10.1017/s0305000905007105 16429710

[B19] Dijkgraaf A. Hartsuiker R. J. Duyck W. (2017). Predicting upcoming information in native-language and non-native-language auditory word recognition. *Biling. Lang. Cogn.* 20 917–930.

[B20] Dijkgraaf A. Hartsuiker R. J. Duyck W. (2019). Prediction and integration of semantics during L2 and L1 listening. *Lang. Cogn. Neurosci.* 34 881–900.

[B21] Döpke S. (2001). “The interplay between language-specific development and crosslinguistic influence,” in *Cross-Linguistic Structures in Simultaneous Bilingualism*, Vol. 21 ed. Döpke S. (Amsterdam: John Benjamins Publishing).

[B22] Dussias P. E. Kroff J. R. V. Tamargo R. E. G. Gerfen C. (2013). When gender and looking go hand in hand: grammatical gender processing in L2 Spanish. *Stud. Second Lang. Acquisit.* 35 353–387. 10.1017/s0272263112000915

[B23] Fenson L. Marchman V. A. Thal D. J. Dale P. S. Reznick J. S. (2007). *MacArthur-Bates Communicative Development Inventories: User’s Guide and Technical Manual.* Baltimore, MD: Brookes.

[B24] Gambi C. Pickering M. J. (2013). Prediction and imitation in speech. *Front. Psychol.* 4:340. 10.3389/fpsyg.2013.00340 23801971 PMC3689255

[B25] Gollan T. H. Montoya R. I. Cera C. Sandoval T. C. (2008). More use almost always means a smaller frequency effect: aging, bilingualism, and the weaker links hypothesis. *J. Mem. Lang.* 58 787–814. 10.1016/j.jml.2007.07.001 19343088 PMC2409197

[B26] Grosjean F. (1989). Neurolinguists, beware! The bilingual is not two monolinguals in one person. *Brain Lang.* 36 3–15. 10.1016/0093-934x(89)90048-5 2465057

[B27] Grüter T. Lew-Williams C. Fernald A. (2012). Grammatical gender in L2: a production or a real-time processing problem? *Sec. Lang. Res.* 28 191–215. 10.1177/0267658312437990 30319164 PMC6181447

[B28] Hansen P. Łuniewska M. Simonsen H. G. Haman E. Mieszkowska K. Kołak J. (2019). Picture-based vocabulary assessment versus parental questionnaires: a cross-linguistic study of bilingual assessment methods. *Intern. J. Biling.* 23 437–456.

[B29] Hansen P. Simonsen H. G. (2016). *PaBiQ, Norsk Versjon: Spørreskjema Om Flerspråklige Barn.* Oslo, NO: Universitet i Oslo.

[B30] Hintz F. Meyer A. S. Huettig F. (2017). Predictors of verb-mediated anticipatory eye movements in the visual world. *J. Exper. Psychol. Learn. Mem. Cogn.* 43:1352. 10.1037/xlm0000388 28287762

[B31] Holmqvist K. Nyström M. Andersson R. Dewhurst R. Jarodzka H. Van de Weijer J. (2011). *Eye Tracking: A Comprehensive Guide to Methods and Measures.* Oxford: Oxford University Press.

[B32] Hopp H. (2015). Semantics and morphosyntax in predictive L2 sentence processing. *Intern. Rev. Appl. Linguist. Lang. Teach.* 53 277–306.

[B33] Huettig F. (2015). Four central questions about prediction in language processing. *Brain Res.* 1626 118–135. 10.1016/j.brainres.2015.02.014 25708148

[B34] Huettig F. Quinlan P. T. McDonald S. A. Altmann G. T. M. (2006). Models of high-dimensional semantic space predict language-mediated eye movements in the visual world. *Acta Psychol.* 121 65–80. 10.1016/j.actpsy.2005.06.002 16098943

[B35] Hulk A. (2001). “Non-selective access and activation in child bilingualism: the syntax,” in *Cross-Linguistic Structures in Simultaneous Bilingualism*, Vol. 21 ed. Döpke S. (Amsterdam: John Benjamins Publishing).

[B36] Ito A. Dunn M. Pickering M. (2017). Effects of language production on prediction: word vs. picture visual world study. *Poster Presented at Architectures and Mechanisms of Language Processing*, Tokyo.

[B37] Kaan E. (2014). Predictive sentence processing in L2 and L1: what is different? *Linguist. Approach. Biling.* 4 257–282.

[B38] Karaca F. Brouwer S. Unsworth S. Huettig F. (2021). “Prediction in bilingual children: the missing piece of the puzzle,” in *Prediction in Second Language Processing and Learning*, eds Kaan E. Grüter T. (Amsterdam: Benjamins).

[B39] Kristoffersen K. Simonsen H. (2012). *Tidlig Spårkutvikling hos Norske Barn: MacArthur-Bates Foreldrerapport for Kommunikativ Utvikling.* Oslo: NOVUS Forlag.

[B40] Lemmerth N. Hopp H. (2019). Gender processing in simultaneous and successive bilingual children: cross-linguistic lexical and syntactic influences. *Lang. Acquisit.* 26 21–45.

[B41] Lemoine-Lardennois C. Alahyane N. Tailhefer C. Collins T. Fagard J. Doré-Mazars K. (2016). Saccadic adaptation in 10–41 month-old children. *Front. Hum. Neurosci.* 10:241. 10.3389/fnhum.2016.00241 27252640 PMC4879146

[B42] Lew-Williams C. Fernald A. (2007). Young children learning Spanish make rapid use of grammatical gender in spoken word recognition. *Psychol. Sci.* 18 193–198. 10.1111/j.1467-9280.2007.01871.x 17444909 PMC3206966

[B43] Lew-Williams C. Fernald A. (2010). Real-time processing of gender-marked articles by native and non-native Spanish speakers. *J. Mem. Lang.* 63 447–464. 10.1016/j.jml.2010.07.003 21076648 PMC2976062

[B44] MacWhinney B. (2008). “A unified model,” in *Handbook of Cognitive Linguistics and Second Language Acquisition*, eds Robinson P. Nick C. Ellis (Hillsdale, NJ: Lawrence Erlbaum Press), 343–371.

[B45] MacWhinney B. (2012). “The logic of the unified model,” in *Handbook of Second Language Acquisition*, eds Susan M. Gass Mackey Alison (New York, NY: Routledge), 211–227.

[B46] Mani N. Daum M. M. Huettig F. (2016). “Proactive” in many ways: Developmental evidence for a dynamic pluralistic approach to prediction. *Q. J. Exper. Psychol.* 69 2189–2201. 10.1080/17470218.2015.1111395 26595092

[B47] Mani N. Huettig F. (2012). Prediction during language processing is a piece of cake—But only for skilled producers. *J. Exper. Psychol. Hum. Percept. Perform.* 38:843. 10.1037/a0029284 22774799

[B48] Martin C. D. Branzi F. M. Bar M. (2018). Prediction is production: the missing link between language production and comprehension. *Sci. Rep.* 8 1–9. 10.1038/s41598-018-19499-4 29348611 PMC5773579

[B49] Meir N. Parshina O. Sekerina I. A. (2020). “The interaction of morphological cues in bilingual sentence processing: an eye-tracking study,” in *Proceedings of the 44th Annual Boston University Conference on Language Development*, eds Brown M. Kohut A. (Sommerville, MA: Cascadilla Press), 376–389.

[B50] Ness T. Meltzer-Asscher A. (2021). Rational adaptation in lexical prediction: the influence of prediction strength. *Front. Psychol.* 12:735849. 10.3389/fpsyg.2021.735849 33935874 PMC8079758

[B51] Pavlenko A. (2009). “Conceptual representation in the bilingual lexicon and second language vocabulary learning,” in *The Bilingual Mental Lexicon: Interdisciplinary Approaches*, ed. Pavlenko A. (Bristol: Multilingual Matters), 125–160.

[B52] Pearson B. Z. Fernández S. C. Oller D. K. (1993). Lexical development in bilingual infants and toddlers: comparison to monolingual norms. *Lang. Learn.* 43 93–120.

[B53] Pickering M. J. Gambi C. (2018). Predicting while comprehending language: a theory and review. *Psychol. Bull.* 144:1002. 10.1037/bul0000158 29952584

[B54] Pickering M. J. Garrod S. (2013). An integrated theory of language production and comprehension. *Behav. Brain Sci.* 36 329–347. 23789620 10.1017/S0140525X12001495

[B55] R Core Team (2021). *R: A Language and Environment for Stastistical Computing [Computer Software].* Vienna: R Fundation for Statistical Computing.

[B56] Rstudio Team (2021). *Rstudio**: Integrated Development for R. [Computer Software].* Boston, MA: Rstudio Team.

[B57] Salverda A. P. Kleinschmidt D. Tanenhaus M. K. (2014). Immediate effects of anticipatory coarticulation in spoken-word recognition. *J. Mem. Lang.* 71 145–163. 10.1016/j.jml.2013.11.002 24511179 PMC3914676

[B58] Stone K. Lago S. Schad D. J. (2020). Divergence point analyses of visual world data: applications to bilingual research. *Biling. Lang. Cogn.* 1–9. 10.1017/S1366728920000607

[B59] The GIMP Team (2018). Gimp 2.8. [Computer Software]. Available online at: https://www.gimp.org

[B60] Trueswell J. C. (2008). Using eye movements as a developmental measure within psycholinguistics. *Lang. Acquis. Lang. Disord.* 44:73.

