# Supplementary material for: Verb-Mediated Prediction in Bilingual Toddlers
Source: Front Psychol. 2021 Nov 11;12:719447. doi: 10.3389/fpsyg.2021.719447 (PMC8631997; doi:10.3389/fpsyg.2021.719447)
Supplement: Supplementary file 1 [file Data_Sheet_1.DOCX]

**Supplementary material**

**Table 1.** Summary of age, gender, language dominance, and CDI vocabulary scores.

| **Participant number** | **Age** | **Gender** | **Norwegian CDI** | **English CDI** |
| --- | --- | --- | --- | --- |
| 1 | 3;0 | M | 588 | 422 |
| 2 | 2;6 | F | 523 | 214 |
| 3 | 2;6 | M | 582 | 133 |
| 4 | 2;6 | M | 606 | 187 |
| 5 | 2;6 | M | 451 | 42 |
| 6 | 2,5 | F | 281 | 178 |
| 7 | 2;6 | F | 456 | 305 |
| 8 | 2;8 | F | 664 | 141 |
| 9 | 3;3 | F | 610 | 56 |
| 10 | 3;1 | M | 484 | 77 |
| 11 | 3;1 | M | 604 | 565 |
| 12 | 3;2 | M | 473 | 122 |
| 13 | 2;9 | M | 609 | 405 |
| 14 | 2;7 | M | 623 | 20 |
| 15 | 2;6 | M | 597 | 244 |
| 16 | 3;3 | F | 639 | N/A* |
| 17 | 2;6 | M | 550 | 370 |

* Parents of one toddler did not fill out the CDI form in English, but reported that though their toddler was dominant in Norwegian, they were almost equally as proficient in English.

**Table 2.** Sentences used in the eye-tracking task. Each sentence pair is presented in the same line, always with the semantically constraining verb first, followed by the neutral verb.

| **Norwegian** | **English** |
| --- | --- |
| Practice trials | |
| Jenta triller den røde ballen ‘The girl rolls the red ball’ | The girl reads the new book |
| Gutten peker på den brune hunden  ‘The boy points at the brown dog’ | The boy smells the blue flower |
| Experimental trials | |
| Gutten *spiser/tar* det grønne eplet  ‘The boy *eats/takes* the green apple’ | The boy *eats/takes* the orange carrot |
| Jenta *bader i/er i* det store badekaret  ‘The girl *bathes in/is in* the big bathtub’ | The girl *bathes/likes* the yellow duck |
| Gutten *sover i/har* den lille senga  ‘The boy *sleeps in/has* the little bed | The boy *sleeps on/likes* the brown couch |
| Jenta *sitter på/bærer på* den blå stolen  ‘The girl *sits on/carries* the blue chair’ | The girl *sits on/looks at* the brown bench |
| Gutten *mater*/*ser på* den store måken  ‘The boy *feeds/looks at* the big seagull’ | The boy *feeds/looks at* the big swan |
| Jenta *åpner/går* til det store vinduet  ‘The girl *opens*/*goes to* the big window’ | The girl *opens/picks up* the big box |
| Gutten *drikker/får* den gule juicen  ‘The boy *drinks/gets* the yellow juice’ | The boy *drinks/gets* the cold milk |
| Jenta *klemmer/holder* den grå bamsen  ‘The girl *hugs/holds* the gray teddy bear’ | The girl *hugs/holds* the big doll |
| Gutten *vanner/finner* den gule solsikken  ‘The boy waters/finds the yellow sunflower’ | The boy *waters/finds* the red rose |
| Jenta *blåser/leker med* de store såpeboblene  ‘The girl *blows/plays with* the big bubbles’ | The girl *blows up/plays with* the red balloon |
| Gutten *kjører/liker* den store bilen  ‘The boy *drives/likes* the big car’ | The boy *drives/wants* the big train |
| Jenta *rir på/har* den brune hesten  ‘The girl *rides on/has* the brown horse’ | The girl *rides on/has* the yellow bike |
| Gutten *skreller/holder* den gule bananen  ‘The boy *peels/holds* the yellow banana’ | The boy *peels/holds* the big orange |
| Jenta *tegner med/tar* den blå fargeblyanten ‘The girl *draws with/takes* the blue color pencil’ | The girl *draws with/takes* the blue crayon |
