# Supplementary material for: Verb-Mediated Prediction in Bilingual Toddlers
Source: Front Psychol. 2021 Nov 11;12:719447. doi: 10.3389/fpsyg.2021.719447 (PMC8631997; doi:10.3389/fpsyg.2021.719447)
Supplement: Supplementary file 2 [file Data_Sheet_2.PDF]

Verb-mediated prediction in bilingual toddlers

Code for the analyses in the manuscript

Ane Theimann, Ekaterina Kuzmina, Pernille Hansen

2021-September-30

1. Data preparation

1.1 Define parameters

```
trial_onset=0
context_onset=1000
verb_onset=3500
noun_onset=5300
trial_offset=8000
time_bin_size=20

trial_onset_bin=ceiling(trial_onset/time_bin_size)
trial_offset_bin=ceiling(trial_offset/time_bin_size)

pred_wind_onset=verb_onset-verb_onset
pred_wind_offset=noun_onset-verb_onset

# Define the period to track after the end of the predictive window to
# capture the divergence point in the neutral condition.
buffer=1700

eye_movement_time_child=300

onsets<-c(trial_onset-verb_onset,
          context_onset-verb_onset,
          verb_onset-verb_onset,
          noun_onset-verb_onset,
          trial_offset-verb_onset)
```

```r
AOI<-c("Target", "Distractor","Fixation", "White_space")

labels_condition<-c(constraining="Constraining condition",
                    neutral="Neutral condition")

labels_language<-c(Norwegian="Norwegian", English="English")

col_experimental<-c("#08519C","#08519C", "gray54", "gray70")

lines_experimental<-c("solid", "twodash", "solid", "twodash")

facet_names<-c(`Norwegian`="Norwegian", `English`="English")

labels_plot<-c("Target"="Target picture",
                "Distractor"="Distractor picture",
                "Fixation"="Fixation picture",
                "White_space"="White space between pictures")
```

1.2 Upload data

```r
prepared_data<-read.delim2("prepared_data.txt", header=TRUE, sep="\t")
prepared_data$X <- NULL
```

2. Pilot study: Adult group

```r
# Compute mean fixation proportions for each participant, trial,
# time, region, condition, and language.
complete_curves_adult<-filter(prepared_data, Group %in% c("Adult")) %>%
  group_by(Participant, Item, Time, Region, Language, Condition) %>%
  summarise(MeanFixation=mean(Value))
```

```
## `summarise()` has grouped output by 'Participant', 'Item', 'Time', 'Region', 'Languag
```

```r
complete_curves_adult$Language<-factor(complete_curves_adult$Language,
                            levels=c("Norwegian", "English"))

complete_curves_adult$Region<-factor(complete_curves_adult$Region, levels=AOI)
```

2.1 Fixation curves for the whole trial length, Figure 2

```
complete_curves_adult_plot<-ggplot(complete_curves_adult,
                                   aes(x=Time,
                                       y=MeanFixation,
                                       colour=Region))+

  geom_vline(xintercept=onsets, linetype="dashed", colour="gray28")+

  stat_summary(fun.data=mean_cl_boot, aes(fill=Region), geom="ribbon",
               alpha=.2, linetype="blank")+

  stat_summary(fun=mean,
               aes(group=Region, linetype=Region), geom="path", size=.7)+

  facet_grid(vars(Condition), vars(Language),
             labeller=labeller(Language=labels_language,
                               Condition=labels_condition))+

  labs(x="Time since verb onset [ms]", y="Fixations to regions")+

  annotate("text", x=onsets[2]-120, y=.85, label="italic(context)",
           parse=TRUE, angle=90, colour="gray28", size=7)+

  annotate("text", x=onsets[3]-120, y=.85, label="italic(verb)",
           parse=TRUE, angle=90, colour="gray28", size=7)+

  annotate("text", x=onsets[4]-120, y=.85, label="italic(noun)",
           parse=TRUE, angle=90, colour="gray28", size=7)+

  scale_x_continuous(breaks=onsets, labels=onsets)+

  scale_y_continuous(labels=scales::percent_format(accuracy=1))+

  scale_colour_manual(values=col_experimental, name="",
                      breaks=AOI, labels=labels_plot)+

  scale_fill_manual(values=col_experimental, name="",
                    breaks=AOI, labels=labels_plot)+

  scale_linetype_manual(values=lines_experimental, name="",
                        breaks=AOI, labels=labels_plot)+

  theme_light()+
```

```
  theme(panel.spacing=unit(1.2, "lines"))+

  theme(text=element_text(size=22, colour="gray28"), legend.position="top")

complete_curves_adult_plot
```

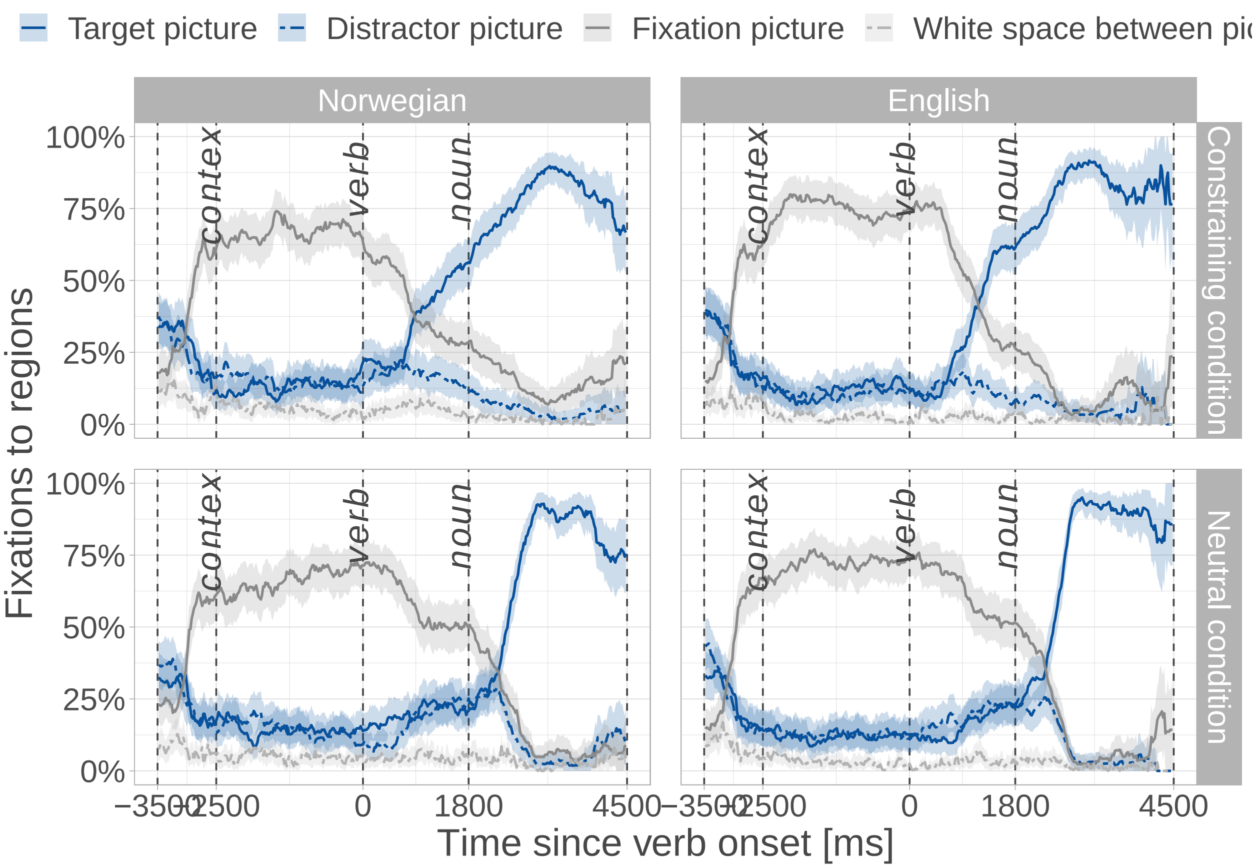


```
jpeg(file=str_replace_all(paste(getwd(), "/Figure_2.jpg"), fixed(" "), ""),
     width=16, height=9, units="in", res=300, pointsize=20)
complete_curves_adult_plot
dev.off()

## pdf
##   2
```

3. Main study: Toddler group

```r
df_analysis<-filter(prepared_data, Group %in% c("Child"))

# Compute mean fixation proportions for each participant, item,
# time, region, condition, and language.
complete_curves_child<-df_analysis %>%
  group_by(Participant, Item, Time, Region, Language, Condition) %>%
  summarise(MeanFixation=mean(Value))
```

```
## `summarise()` has grouped output by 'Participant', 'Item', 'Time', 'Region', 'Languag
```

```r
complete_curves_child$Language<-factor(complete_curves_child$Language,
                                       levels=c("Norwegian",
                                                "English"))

complete_curves_child$Region<-factor(complete_curves_child$Region,
                                     levels=AOI)
```

3.1 Fixation curves for the whole trial length, Figure 3

```r
complete_curves_child_plot<-ggplot(complete_curves_child,
                                   aes(x=Time,
                                       y=MeanFixation,
                                       colour=Region))+

  geom_vline(xintercept=onsets, linetype="dashed", colour="gray28")+

  stat_summary(fun.data=mean_cl_boot, aes(fill=Region),
               geom="ribbon", alpha=.2, linetype="blank")+

  stat_summary(fun=mean, aes(group=Region, linetype=Region),
               geom="path", size=.7)+

  facet_grid(vars(Condition), vars(Language),
             labeller=labeller(Language=labels_language,
                               Condition=labels_condition))+

  labs(x="Time since verb onset [ms]",
       y="Fixations to regions")+

  annotate("text", x=onsets[2]-120, y=.75, label="italic(context)",
           parse=TRUE, angle=90, colour="gray28", size=7)+
```

```r
  annotate("text", x=onsets[3]-120, y=.75, label="italic(verb)",
           parse=TRUE, angle=90, colour="gray28", size=7)+

  annotate("text", x=onsets[4]-120, y=.75, label="italic(noun)",
           parse=TRUE, angle=90, colour="gray28", size=7)+

  scale_x_continuous(breaks=onsets, labels=onsets)+

  scale_y_continuous(labels=scales::percent_format(accuracy=1))+

  scale_colour_manual(values=col_experimental,
                      name="", breaks=AOI, labels=labels_plot)+

  scale_fill_manual(values=col_experimental,
                    name="", breaks=AOI, labels=labels_plot)+

  scale_linetype_manual(values=lines_experimental,
                        name="", breaks=AOI, labels=labels_plot)+

  theme_light()+

  theme(panel.spacing=unit(1.2, "lines"))+

  theme(text=element_text(size=22, colour="gray28"), legend.position="top")

complete_curves_child_plot
```

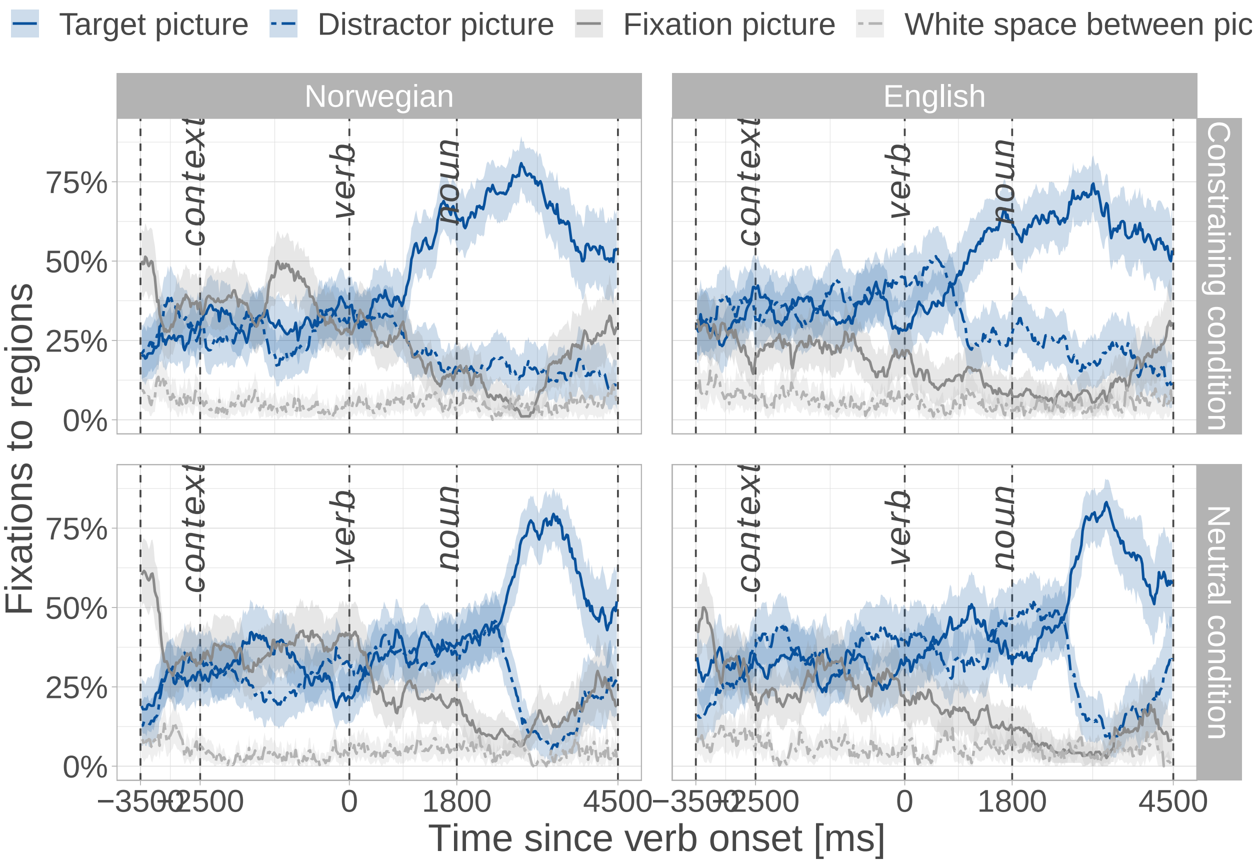


```
jpeg(file=str_replace_all(paste(getwd(), "/Figure_3.jpg"), fixed(" "), ""),
     width=16, height=9, units="in", res=300, pointsize=20)
complete_curves_child_plot
dev.off()
```

```
## pdf
##   2
```

3.2 GLMM in Norwegian

3.2.1 Prepare data for fixation curves in the critical predicitve window

```
# Create dependent variables
df_after_verb_nor<-df_analysis %>%
  filter(Language=="Norwegian") %>%
  # Extract the critical window
  subset(between(Time, pred_wind_onset,
                 pred_wind_offset + buffer + 500)) %>%
  # Create mean and sum of fixations by participant, item,
```

```
  # time bin, region, and experimental conditions
  group_by(Participant, Item, Time, Region, Condition) %>%
  summarise(MeanFixation=mean(Value),
            SumFixation=sum(Value),
            NFixation=length(Value)) %>%
  # Extract the two regions of interest
  subset(Region %in% c("Target", "Distractor")) %>%
  # Discard unwanted factor levels
  droplevels()
```

```
## `summarise()` has grouped output by 'Participant', 'Item', 'Time', 'Region'. You can
```

```
# Reorder factor levels, add nicer labels
df_after_verb_nor$Condition<-factor(df_after_verb_nor$Condition,
                      levels=c("constraining", "neutral"))

labels_condition<-c(constraining="Constraining condition",
                      neutral="Neutral condition")


# Create plot with Time on the x-axis and mean fixations on the y-axis
p_child_nor<-
  ggplot(df_after_verb_nor, aes(x=Time, y=MeanFixation))+
  # Plot the mean fixation proportions by region as a line
  stat_summary(fun=mean, geom="path",
               aes(group=Region, colour=Region, linetype=Region),
               size=.7)+
  # Add confidence intervals
  stat_summary(fun.data=mean_cl_boot, geom="ribbon",
               aes(fill=Region), alpha=.2)+
  # Create a separate panel for each condition
  facet_grid(vars(Condition), scales="free_x",
             labeller=labeller(Condition=labels_condition))+

  theme(text=element_text(size=22, colour="gray28"),
        legend.position="top", legend.title=element_blank(),
        panel.spacing=unit(1.2, "lines"))+

  scale_colour_manual(values=c("#08519C", "#08519C"),
                      breaks=c("Target", "Distractor"),
                      labels=c("Target", "Distractor"))+

  scale_fill_manual(values=c("#08519C", "#08519C"),
                    breaks=c("Target", "Distractor"),
```

```
                    labels=c("Target", "Distractor"))+

  guides(colour=guide_legend(keywidth=1, keyheight=1,
                             default.unit="cm"),
         fill=guide_legend(keywidth=1, keyheight=1,
                           default.unit="cm"),
         linetype=guide_legend(keywidth=1, keyheight=1,
                               efault.unit="cm"))+

  scale_linetype_manual(values=c("solid","dotdash"),
                        breaks=c("Target", "Distractor"),
                        labels=c("Target", "Distractor"))+

  scale_y_continuous(breaks=seq(0, 1, by=.25),
                     labels=scales::percent_format(accuracy=1))+

  scale_x_continuous(breaks=seq(pred_wind_onset,
                                pred_wind_offset+buffer+500, 400),
                     labels=seq(pred_wind_onset,
                                pred_wind_offset+buffer+500, 400))+

  labs(x="Time since verb onset [ms]",
       y="Fixations to regions")+

  geom_vline(xintercept =noun_onset-verb_onset, linetype="dashed",
             size=1, colour="gray28")+

  geom_vline(xintercept =verb_onset-verb_onset, linetype="dashed",
             size=1, colour="gray28")+

  annotate("text", label="italic(verb)",
           y=.75, x=verb_onset-verb_onset-40,
           parse=TRUE, angle=90, colour="gray28", size=7)+

  annotate("text", label="italic(noun)", parse=TRUE,
           y=.75, x=noun_onset-verb_onset-40,
           angle=90, size=7, colour="gray28")+

  theme_light()

p_child_nor
```

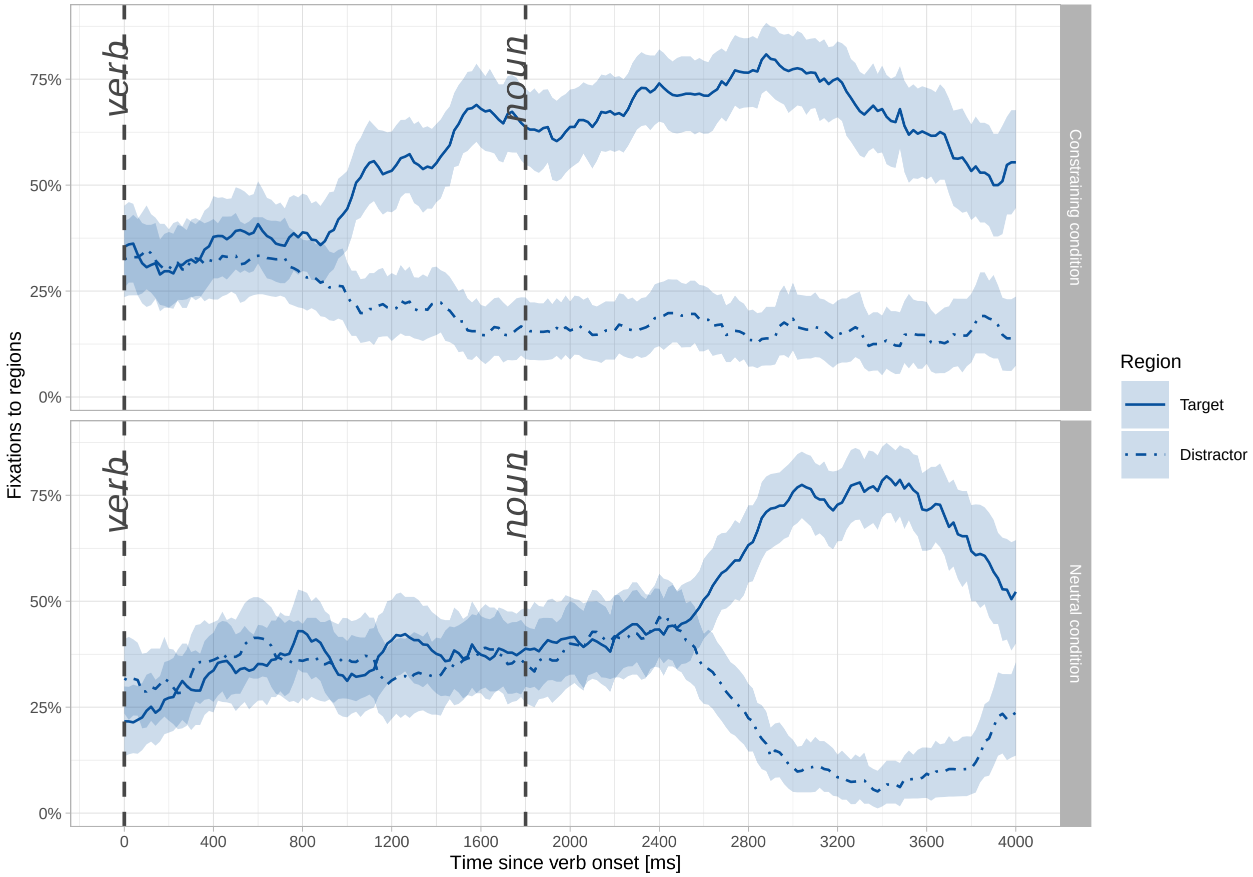

3.2.2 GLMM

```
# Extract the critical predictive window
df_stat_child_nor<-df_analysis %>%
  filter(Language=="Norwegian") %>%
  filter(between(Time, pred_wind_onset + eye_movement_time_child,
                 pred_wind_offset + eye_movement_time_child + buffer))

# Apply statistical test for each time bin
p_table_nor<-df_stat_child_nor %>%
  # Select only rows where a fixation occurred
  filter(Value==1) %>%
  # Select only the two regions of interest
  filter(Region %in% c("Target", "Distractor")) %>%
  # Label fixations that were on the target as 1 and as 0 otherwise,
  mutate(pTarget=ifelse(Region=="Target", 1, 0)) %>%
  # Prepare to test for each condition in each time bin
  group_by(Condition, Time) %>%
  # Apply logistic regression and extract z-values
```

```r
  summarise(z=summary(lme4::glmer(pTarget ~ 1 +
        (1|Participant)+ (1|Item), family="binomial",
        control=lme4::glmerControl(calc.derivs=FALSE)))$coefficients[1,3]) %>%
  # Add p-values
  mutate(p=pnorm(-abs(z))*2)
```

```
## Registered S3 methods overwritten by 'lme4':
##   method                          from
##   cooks.distance.influence.merMod car
##   influence.merMod                car
##   dfbeta.influence.merMod         car
##   dfbetas.influence.merMod        car
```

```
## `summarise()` has grouped output by 'Condition'. You can override using the `.groups`
```

```r
# Define alpha level and calculate the Bonferroni correction
alpha          <-0.05
N_tests        <-length(unique(df_stat_child_nor$Time))
alpha_corrected<-alpha/N_tests
```

```r
uncorr_nor<-p_table_nor %>%
  # Extract positive z-scores ("target advantage") and significant p-values
  filter(z > 0, p < alpha) %>%
  group_by(Condition) %>%
  # Extract the earliest significant p-value and its time bin
  slice(1)

uncorr_nor
```

Get uncorrected for multiple comparisons estimates

```
## # A tibble: 2 x 4
## # Groups:   Condition [2]
##   Condition     Time     z      p
##   <chr>        <int> <dbl>  <dbl>
## 1 constraining   920  2.14 0.0324
## 2 neutral       2640  2.08 0.0379
```

```r
bnf_nor<-p_table_nor %>%
  filter(z > 0, p < alpha_corrected) %>%
  group_by(Condition) %>%
  slice(1)

bnf_nor
```

Apply Bonferroni correction

```
## # A tibble: 2 x 4
## # Groups:   Condition [2]
##   Condition     Time     z         p
##   <chr>        <int> <dbl>     <dbl>
## 1 constraining  1560  3.67 0.000243
## 2 neutral       2840  4.20 0.0000270
```

```r
fdr_nor<-p_table_nor %>%
  mutate(p_fdr=stats::p.adjust(p, method="BY", n=length(p))) %>%
  filter(z > 0, p_fdr < alpha) %>%
  group_by(Condition) %>%
  slice(1)

fdr_nor
```

Apply FDR control correction

```
## # A tibble: 2 x 5
## # Groups:   Condition [2]
##   Condition     Time     z       p  p_fdr
##   <chr>        <int> <dbl>   <dbl>  <dbl>
## 1 constraining  1020  3.01 0.00264 0.0262
## 2 neutral       2720  3.28 0.00104 0.0493
```

```r
# Add the estimates to the base plot
nor_plot<-p_child_nor +
  # FDR-corrected onset
```

```
geom_point(data=subset(df_after_verb_nor, Condition=="constraining"),
      aes(y=.38, x=fdr_nor$Time[fdr_nor$Condition=="constraining"]),
      size=5, stroke=2, shape=17, color="gray28")+

geom_point(data=subset(df_after_verb_nor, Condition=="neutral"),
        aes(y=.38, x=fdr_nor$Time[fdr_nor$Condition=="neutral"]),
        size=5, stroke=2, shape=17, color="gray28")+

geom_vline(xintercept=c(fdr_nor$Time[fdr_nor$Condition=="constraining"],
                      fdr_nor$Time[fdr_nor$Condition=="neutral"]),
         linetype="dashed", colour="gray28", size=0.2)+

theme(text=element_text(size=22, colour="gray28"),
      #legend.position="bottom",
      axis.title.x = element_blank(),
      plot.title = element_text(hjust = 0.5, size = 25, face = "bold"))+

ggtitle("Norwegian")

# Uncorrected onset
#geom_point(data=subset(df_after_verb_nor, Condition=="constraining"),
#         aes(y=.58,
#             x=uncorr_nor$Time[uncorr_nor$Condition=="constraining"]),
#         size=5, shape=1, color="gray28")+
#geom_point(data=subset(df_after_verb_nor, Condition=="neutral"),
#         aes(y=.58, x=uncorr_nor$Time[uncorr_nor$Condition=="neutral"]),
#         size=5, shape=1, color="gray28")+
#
#geom_vline(xintercept=c(uncorr_nor$Time[uncorr_nor$Condition=="constraining"],
#                       uncorr_nor$Time[uncorr_nor$Condition=="neutral"]),
#           linetype="dashed", colour="gray28", size=0.2)+

# Bonferroni-corrected onset
#geom_point(data=subset(df_after_verb_nor, Condition=="constraining"),
#         aes(y=.48, x=bnf_nor$Time[bnf_nor$Condition=="constraining"]),
#         size=5, shape=15, color="gray28")+

#geom_point(data=subset(df_after_verb_nor,  Condition=="neutral"),
#         aes(y=.48, x=bnf_nor$Time[bnf_nor$Condition=="neutral"]),
#         size=5, shape=15, color="gray28")+

#geom_vline(xintercept=c(bnf_nor$Time[bnf_nor$Condition=="constraining"],
#                       bnf_nor$Time[bnf_nor$Condition=="neutral"]),
#           linetype="dashed", colour="gray28", size=0.2)
```

```
nor_plot
```

Add divergence point estimates to the fixation curves

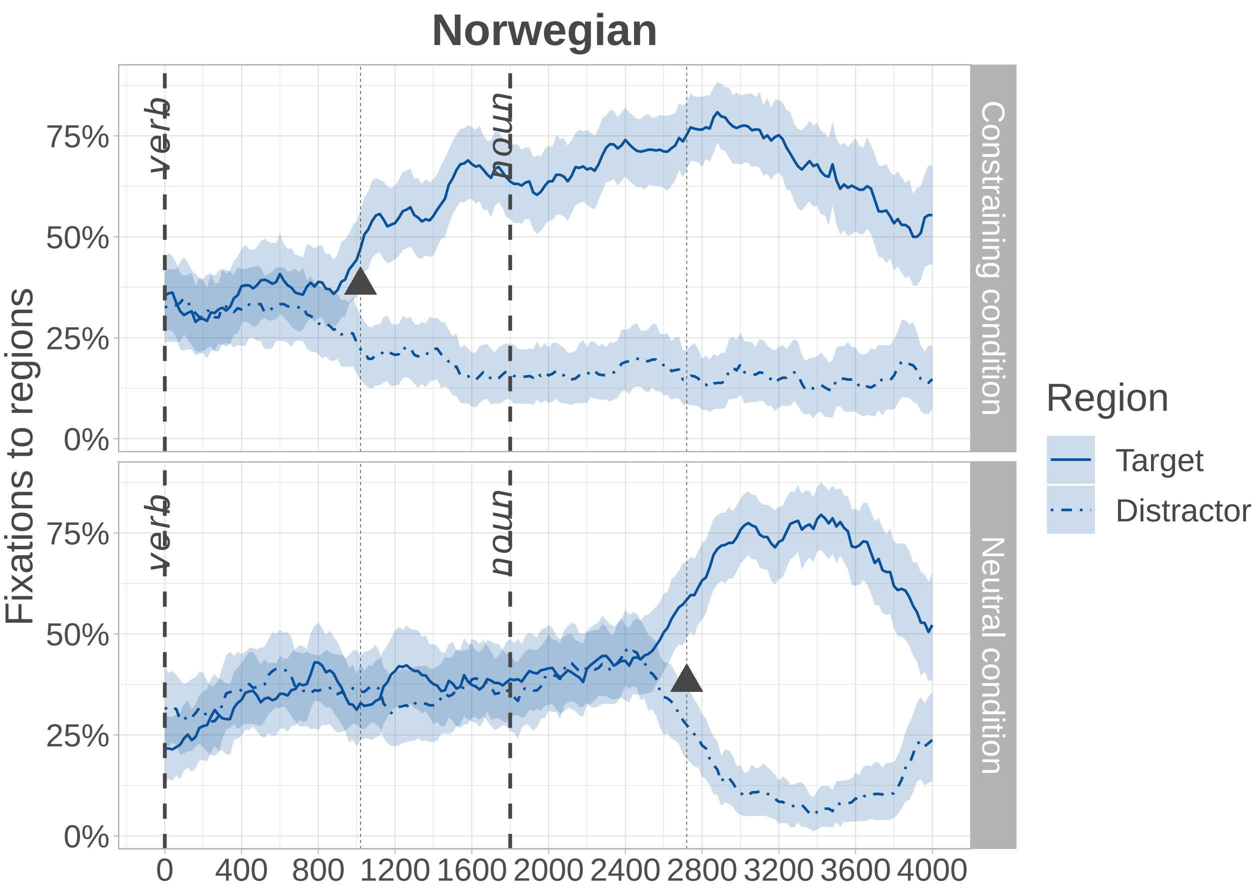

3.3 GLMM in English

3.3.1 Prepare data for fixation curves in the critical predicitve window

```
# Create dependent variables
dat_plot_child_eng<-df_analysis %>%
    filter(Language=="English") %>%
  # Extract the critical window
  subset(between(Time, pred_wind_onset,
                 pred_wind_offset + buffer + 500)) %>%
  # Create mean and sum of fixations by participant, item,
  # time bin, region, and experimental conditions
  group_by(Participant, Item, Time, Region,Condition) %>%
```

```
  summarise(MeanFixation=mean(Value),
            SumFixation=sum(Value),
            NFixation=length(Value)) %>%
  # Extract the two regions of interest
  subset(Region %in% c("Target", "Distractor")) %>%
  # Discard unwanted factor levels
  droplevels()
```

```
## `summarise()` has grouped output by 'Participant', 'Item', 'Time', 'Region'. You can
```

```
# Reorder factor levels, add nicer labels
dat_plot_child_eng$Condition<-factor(dat_plot_child_eng$Condition,
                      levels=c("constraining", "neutral"))

labels_condition<-c(constraining="Constraining condition",
                      neutral="Neutral condition")



# Create plot with Time on the x-axis and mean fixations on the y-axis
p_child_eng<-ggplot(dat_plot_child_eng, aes(x=Time, y=MeanFixation))+
  # Plot the mean fixation proportions by region as a line
  stat_summary(fun=mean, geom="path",
               aes(group=Region, colour=Region, linetype=Region),
               size=.7)+
  # Add confidence intervals
  stat_summary(fun.data=mean_cl_boot, geom="ribbon",
               aes(fill=Region), alpha=.2)+
  # Create a separate panel for each condition
  facet_grid(vars(Condition),  scales="free_x",
             labeller=labeller(Condition=labels_condition))+

  theme(text=element_text(size=22, colour="gray28"),
        legend.title=element_blank(),
        panel.spacing=unit(1.7, "lines"))+

  scale_colour_manual(values=c("#08519C", "#08519C"),
                      breaks=c("Target", "Distractor"),
                      labels=c("Target", "Distractor"))+

  scale_fill_manual(values=c("#08519C", "#08519C"),
                    breaks=c("Target", "Distractor"),
                    labels=c("Target", "Distractor"))+

  guides(colour=FALSE, fill=FALSE, linetype=FALSE)+
```

```r
  scale_linetype_manual(values=c("solid","dotdash"),
                        breaks=c("Target", "Distractor"),
                        labels=c("Target", "Distractor"))+

  scale_y_continuous(breaks=seq(0, 1, by=.25),
                     labels=scales::percent_format(accuracy=1))+

  scale_x_continuous(breaks=seq(pred_wind_onset,
                               pred_wind_offset + buffer + 500, 400),
                     labels=seq(pred_wind_onset,
                               pred_wind_offset + buffer + 500, 400))+

  labs(x="Time since verb onset [ms]",
       y="Fixations to regions")+

  geom_vline(xintercept=noun_onset-verb_onset, linetype="dashed",
             size=1, colour="gray28")+

  geom_vline(xintercept=verb_onset-verb_onset, linetype="dashed",
             size=1, colour="gray28")+

  annotate("text", label="italic(verb)",
           y=.75, x=verb_onset-verb_onset-40,
           parse=TRUE, angle=90, colour="gray28", size=7)+

  annotate("text", label="italic(noun)", parse=TRUE,
           y=.75, x=noun_onset-verb_onset-40,
           angle=90, size=7, colour="gray28")+

  theme_light()

p_child_eng
```

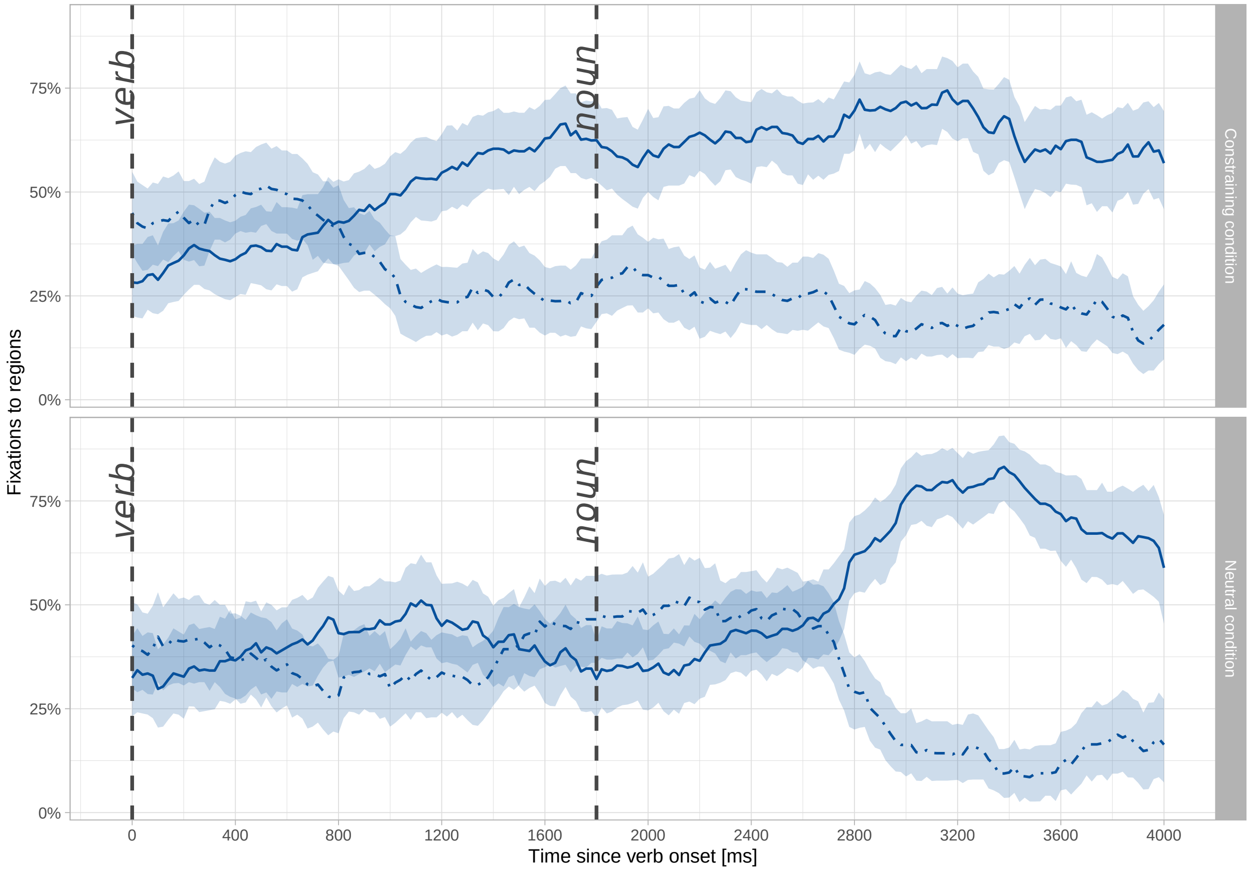

3.3.2 GLMM

```
# Extract the critical predictive window.
df_stat_child_eng<-df_analysis %>%
  filter(Language=="English") %>%
  filter(between(Time, pred_wind_onset + eye_movement_time_child,
                 pred_wind_offset + eye_movement_time_child + buffer))

# Apply statistical test for each time bin.
p_table_eng<-df_stat_child_eng %>%
  # Select only rows where a fixation occurred
  filter(Value==1) %>%
  # Select only the two regions of interest
  filter(Region %in% c("Target", "Distractor")) %>%
  # Label fixations that were on the target as 1 and as 0 otherwise
  mutate(pTarget=ifelse(Region=="Target", 1, 0)) %>%
  # Test for each condition at each time bin
  group_by(Condition, Time) %>%
  # Apply logistic regression and extract z-values
  summarise(z=summary(lme4::glmer(pTarget ~ 1 +
```

```r
        (1|Participant)+ (1|Item), family="binomial",
        control=lme4::glmerControl(calc.derivs=FALSE)))$coefficients[1,3]) %>%
  # Add p-values
  mutate(p=pnorm(-abs(z))*2)
```

```
## `summarise()` has grouped output by 'Condition'. You can override using the `.groups`
```

```r
# Define alpha level and calculate the Bonferroni correction
alpha                 <-0.05
N_tests_eng           <-length(unique(df_stat_child_eng$Time))
alpha_corrected_eng<-alpha/N_tests_eng
```

```r
uncorr_eng<-p_table_eng %>%
  # Extract positive z-scores ("target advantage") and significant p-values
  filter(z > 0, p < alpha) %>%
  # Group results by condition group
  group_by(Condition) %>%
  # Extract the earliest significant p-value and its timepoint
  slice(1)

uncorr_eng
```

Get uncorrected for multiple comparisons estimates

```
## # A tibble: 2 x 4
## # Groups:   Condition [2]
##   Condition     Time     z      p
##   <chr>        <int> <dbl>  <dbl>
## 1 constraining  1300  1.96 0.0495
## 2 neutral       2780  2.37 0.0178
```

```r
bnf_eng<-p_table_eng %>%
  filter(z > 0, p < alpha_corrected_eng) %>%
  group_by(Condition) %>%
  slice(1)

bnf_eng
```

Apply Bonferroni correction

```
## # A tibble: 2 x 4
## # Groups:   Condition [2]
##   Condition     Time     z        p
##   <chr>        <int> <dbl>    <dbl>
## 1 constraining  2560  3.66 0.000249
## 2 neutral       3020  3.64 0.000272
```

```
fdr_eng<-p_table_eng %>%
  mutate(p_fdr_eng=stats::p.adjust(p, method="BY", n=length(p))) %>%
  filter(z > 0, p_fdr_eng < alpha) %>%
  group_by(Condition) %>%
  slice(1)

fdr_eng
```

Apply FDR control correction

```
## # A tibble: 2 x 5
## # Groups:   Condition [2]
##   Condition     Time     z        p p_fdr_eng
##   <chr>        <int> <dbl>    <dbl>     <dbl>
## 1 constraining  1620  2.95 0.00322     0.0453
## 2 neutral       2960  3.49 0.000484    0.0314
```

```
eng_plot<-p_child_eng+
  # FDR-corrected onset
  geom_point(data=subset(dat_plot_child_eng, Condition=="constraining"),
          aes(y=.38, x=fdr_eng$Time[fdr_eng$Condition=="constraining"]),
          size=5, stroke=2, shape=17, color="black")+

  geom_point(data=subset(dat_plot_child_eng, Condition=="neutral"),
          aes(y=.38, x=fdr_eng$Time[fdr_eng$Condition=="neutral"]),
          size=5, stroke=2, shape=17, color="black")+

  geom_vline(xintercept=c(fdr_eng$Time[fdr_eng$Condition=="constraining"],
                          fdr_eng$Time[fdr_eng$Condition=="neutral"]),
           linetype="dashed", colour="gray28", size=0.2)+

  theme(text=element_text(size=22, colour="gray28"),
```

```r
        legend.position="bottom",
        axis.title.x = element_blank(),
        plot.title = element_text(hjust = 0.5, size = 25, face = "bold"))+

  ggtitle("English")

  # Uncorrected onset
  #geom_point(data=subset(dat_plot_child_eng, Condition=="constraining"),
  #         aes(y=.58,
  #             x=uncorr_eng$Time[uncorr_eng$Condition=="constraining"]),
  #         size=5, shape=1, color="gray28")+

  #geom_point(data=subset(dat_plot_child_eng, Condition=="neutral"),
  #         aes(y=.58,
  #             x=uncorr_eng$Time[uncorr_eng$Condition=="neutral"]),
  #         size=5, shape=1, color="gray28")+

  #geom_vline(xintercept=c(uncorr_eng$Time[uncorr_eng$Condition=="constraining"],
  #                        uncorr_eng$Time[uncorr_eng$Condition=="neutral"]),
  #            linetype="dashed", colour="gray28", size=0.2)+

  # Bonferroni-corrected onset
  #geom_point(data=subset(dat_plot_child_eng, Condition=="constraining"),
  #         aes(y=.48, x=bnf_eng$Time[bnf_eng$Condition=="constraining"]),
  #         size=5, shape=15, color="gray28")+

  #geom_point(data=subset(dat_plot_child_eng,  Condition=="neutral"),
  #         aes(y=.48, x=bnf_eng$Time[bnf_eng$Condition=="neutral"]),
  #         size=5, shape=15, color="gray28")+

  #geom_vline(xintercept=c(bnf_eng$Time[bnf_eng$Condition=="constraining"],
  #                        bnf_eng$Time[bnf_eng$Condition=="neutral"]),
  #            linetype="dashed", colour="gray28", size=0.2)

eng_plot
```

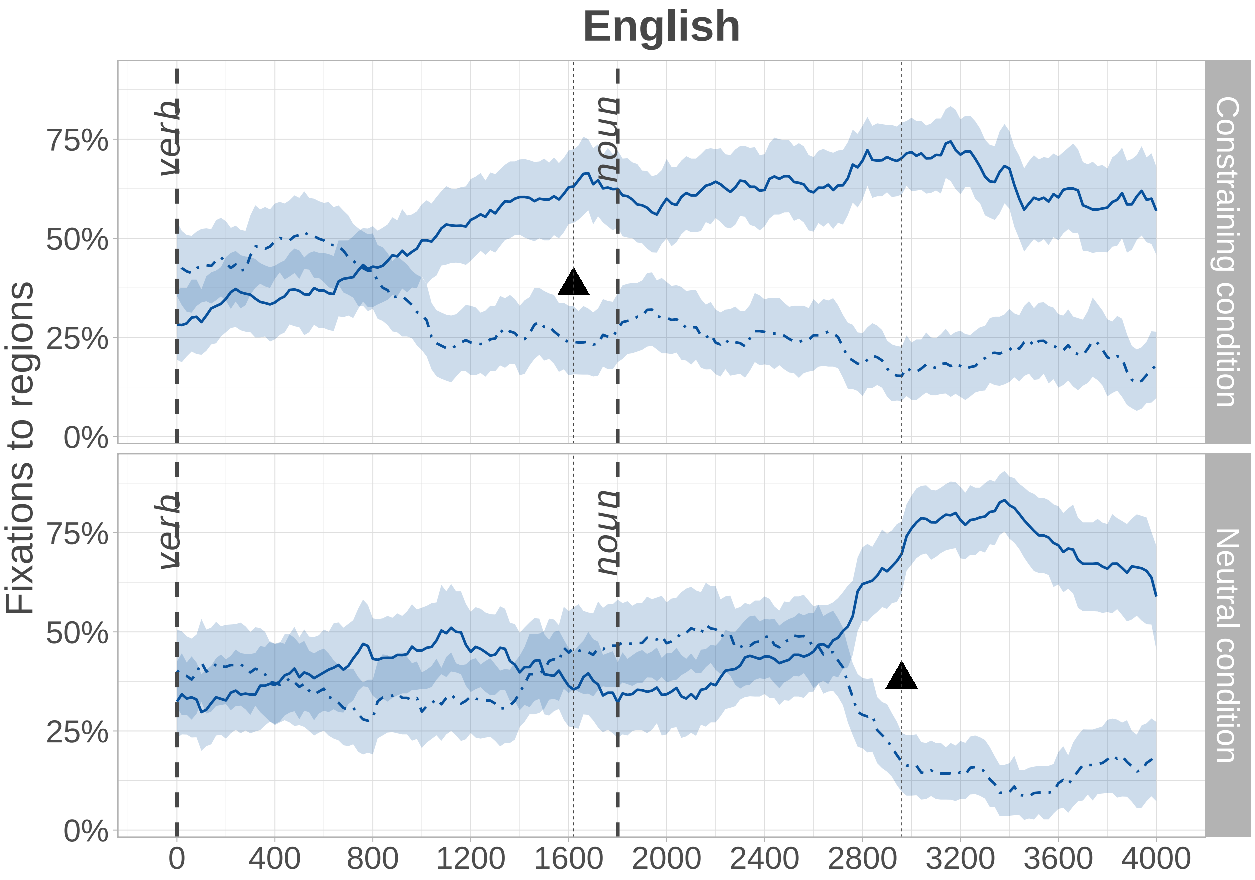


```r
figure_4<-ggarrange(nor_plot, eng_plot,
                    ncol=1, nrow=2,
                    common.legend = TRUE, legend="bottom")

figure_4
```

Put estimates from both languages together, Figure 4

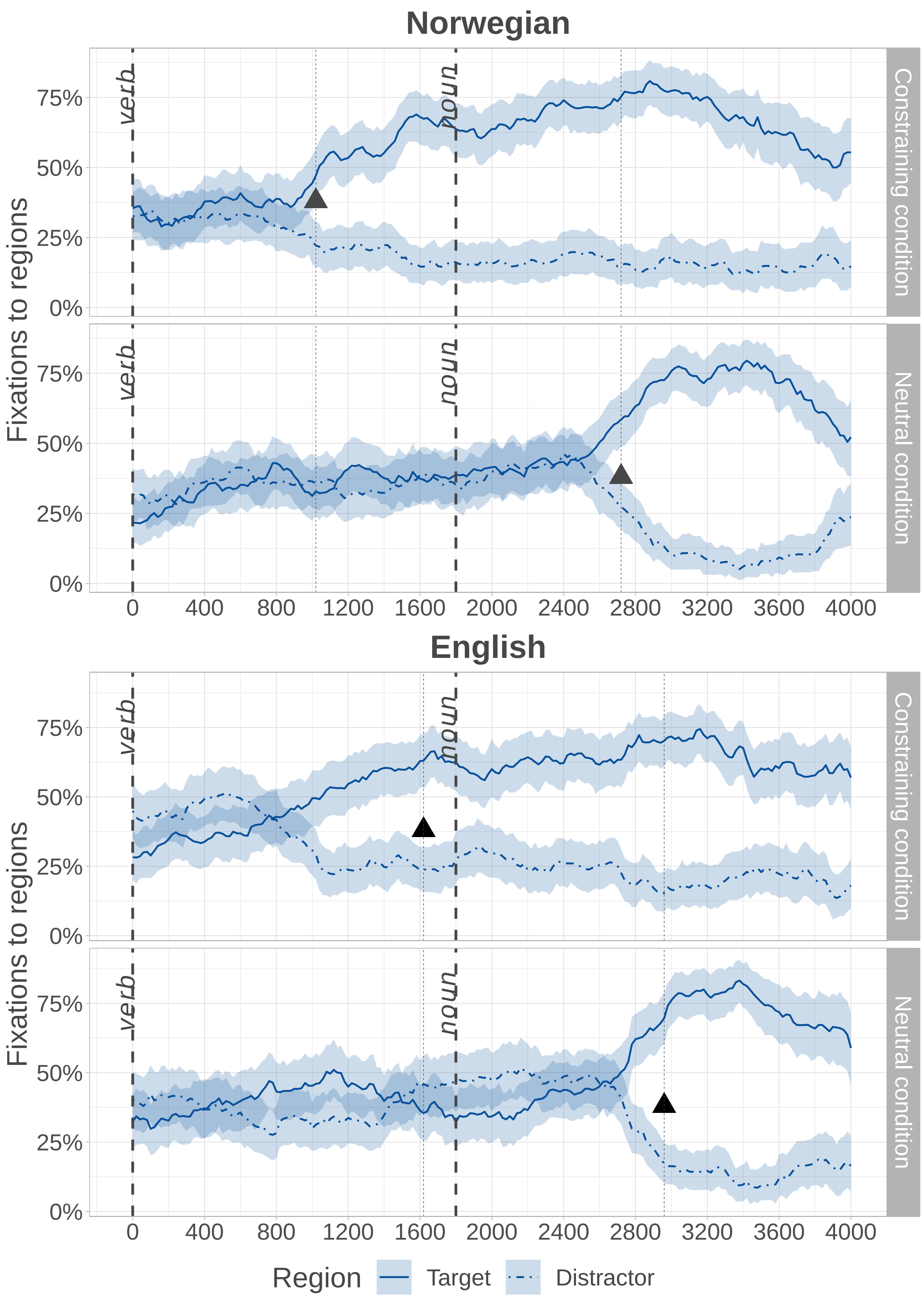

Norwegian
Fixations to regions
75%
50%
25%
0%
verb
noun
Constraining condition
Neutral condition
0
400
800
1200
1600
2000
2400
2800
3200
3600
4000
English
Fixations to regions
Region
Target
Distractor

```
jpeg(file=str_replace_all(paste(getwd(), "/Figure_4.jpg"), fixed(" "), ""),
     width=16, height=14, units="in", res=1000, pointsize=20)
figure_4
dev.off()
```

```
## pdf
##   2
```

3.4 Get the relationship between expressive vocabulary and predicitve ability

```
# Upload vocabulary data
vocabulary_data<-read.csv("vocab.csv", header=TRUE, sep=";")
vocabulary_data<-rename(vocabulary_data,c('Language'='language',
                                            'Participant'='participant'))

data_nor_eng<-rbind(df_stat_child_eng, df_stat_child_nor) %>%
    # Subset only the critical predictive window
  subset(between(Time,
                 pred_wind_onset + eye_movement_time_child,
                 pred_wind_offset + eye_movement_time_child)) %>%
  filter(Region=="Target")

timebins_count<-length(unique(data_nor_eng$Time))

cor_data<-data_nor_eng %>%
  group_by(Participant, Language, Condition, Time) %>%
  summarise(Mean_fixation=mean(Value)) %>%
  group_by(Participant, Language, Condition) %>%
  summarise(Mean_fixation=mean(Mean_fixation)) %>%
  spread(Condition, Mean_fixation)
```

```
## `summarise()` has grouped output by 'Participant', 'Language', 'Condition'. You can o

## `summarise()` has grouped output by 'Participant', 'Language'. You can override using
```

```
cor_data<-rename(cor_data, c('Language'='Language'))

cor_data$constr_to_neutral_diff<-cor_data$constraining - cor_data$neutral

# Merge vocabulary together with prediction data
```

```r
cor_data<-merge(cor_data, vocabulary_data,
                    by=c("Participant", "Language"))

cor_data$Language<-factor(cor_data$Language,
                              levels=c("Norwegian", "English"))

cor_nor <-cor_data[cor_data$Language=="Norwegian", ]
cor_eng <-cor_data[cor_data$Language=="English", ]
```

3.4.1 Predictive ability vs. expressive vocabularies, Figure 5

```r
cor_prod_pred <-
  ggplot(cor_data,
            aes(x=cdi,y=constr_to_neutral_diff))+
            theme(legend.position="none")+
            facet_wrap(~Language)+
            geom_point(size=5)+
            geom_smooth(method=lm)+
            xlab("Productive vocabulary in each language")+
            ylab("Predictive ability")+
            theme_light()+
            theme(text=element_text(size=22, colour="gray28"),
            panel.spacing=unit(1.2, "lines"))+
            scale_x_continuous(breaks=seq(0, 600, 100),
            labels=seq(0, 600, 100))

# Display the plot
cor_prod_pred
```

```
## `geom_smooth()` using formula 'y ~ x'
```

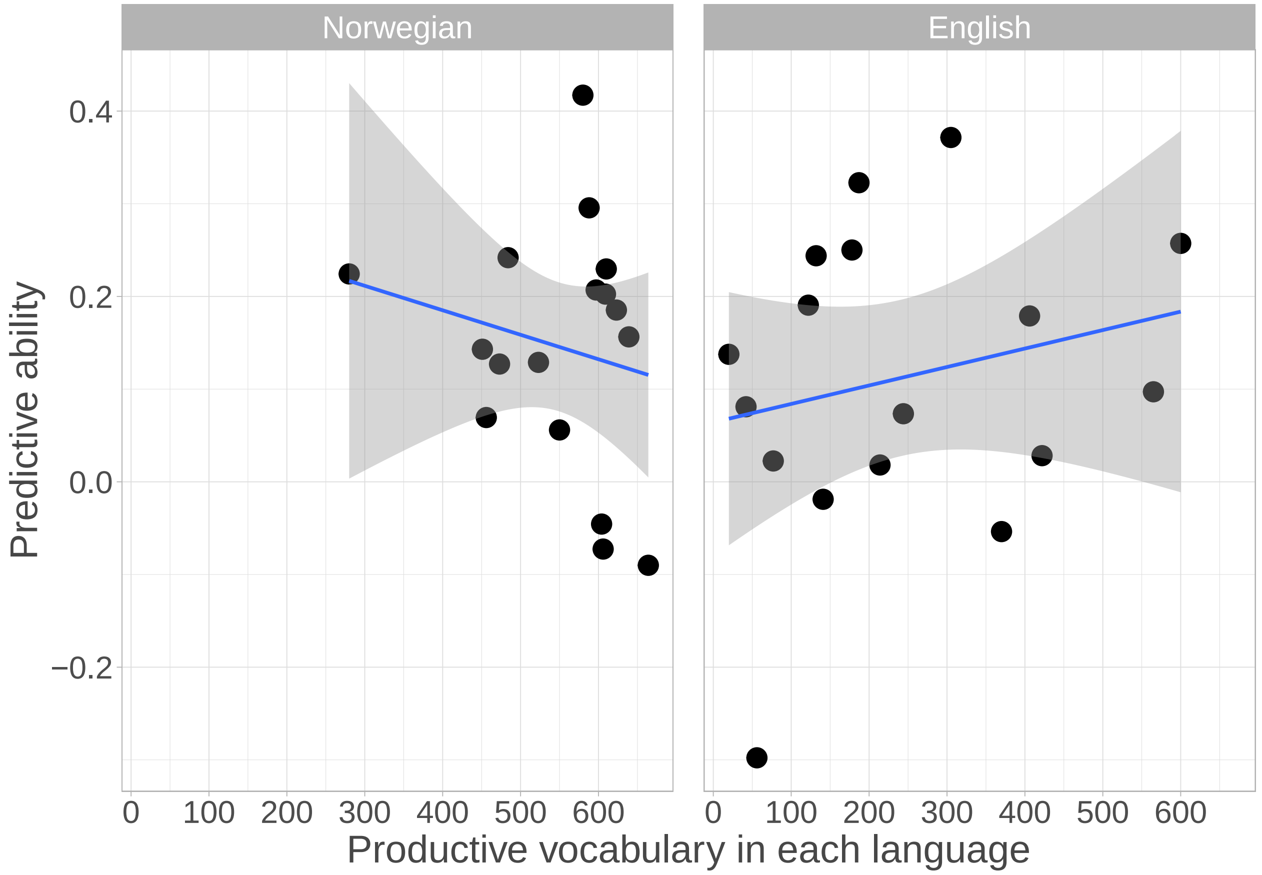


```
jpeg(file=str_replace_all(paste(getwd(), "/Figure_5.jpg"), fixed(" "), ""),
     width=14, height=6, units="in", res=300)
cor_prod_pred
```

```
## `geom_smooth()` using formula 'y ~ x'
```

```
dev.off()
```

```
## pdf
##   2
```

```
cor.test(cor_nor$constr_to_neutral_diff,
         cor_nor$cdi,
         method="spearman")
```

```
##
##  Spearman's rank correlation rho
##
## data:  cor_nor$constr_to_neutral_diff and cor_nor$cdi
```

```
## S = 930, p-value = 0.592
## alternative hypothesis: true rho is not equal to 0
## sample estimates:
##        rho
## -0.1397059
```

```
cor.test(cor_eng$constr_to_neutral_diff,
         cor_eng$cdi,
         method="spearman")
```

```
##
##  Spearman's rank correlation rho
##
## data:  cor_eng$constr_to_neutral_diff and cor_eng$cdi
## S = 662, p-value = 0.4668
## alternative hypothesis: true rho is not equal to 0
## sample estimates:
##       rho
## 0.1887255
```

3.4.2 Predictive ability vs. total expressive vocabulary, Figure 6

```
cor_prod_pred_total <-
  ggplot(cor_data,
            aes(x=cdi_total,y=constr_to_neutral_diff))+
            theme(legend.position="none")+
            facet_wrap(~Language)+
            geom_point(size=5)+
            geom_smooth(method=lm)+
            xlab("Total productive vocabulary")+
            ylab("Predictive ability")+
            theme_light()+
            theme(text=element_text(size=22, colour="gray28"),
            panel.spacing=unit(1.2, "lines"))+
            scale_x_continuous(breaks=seq(0, 2000, 100),
            labels=seq(0, 2000, 100))

# Display the plot
cor_prod_pred_total
```

```
## `geom_smooth()` using formula 'y ~ x'
```

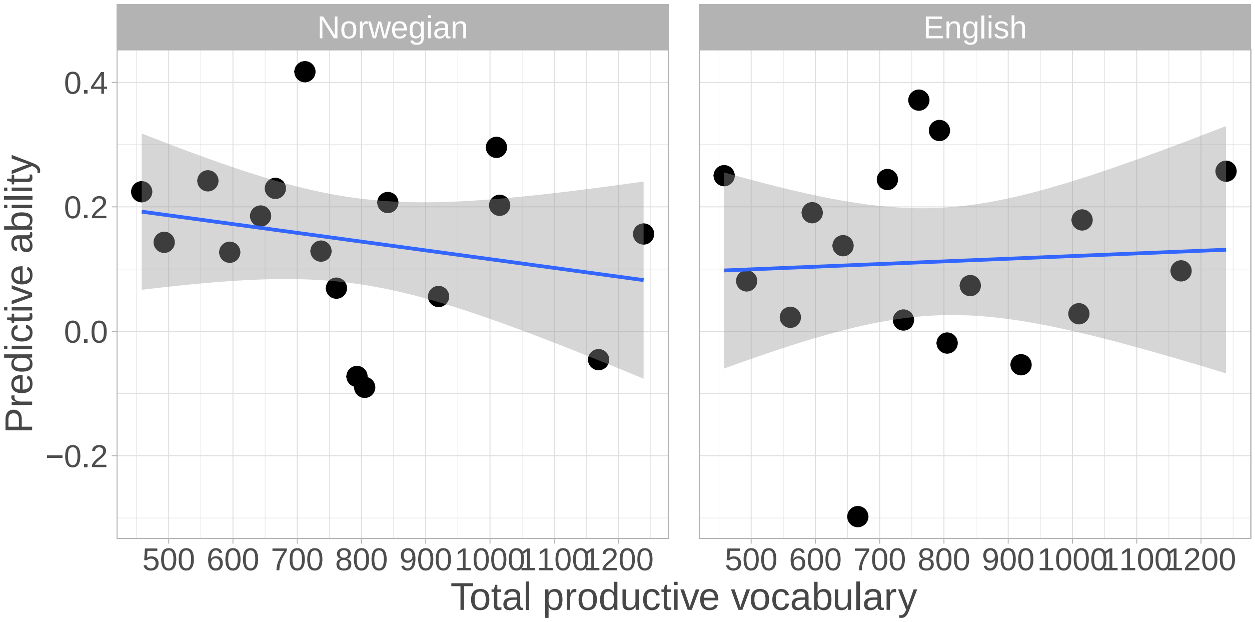


```
jpeg(file=str_replace_all(paste(getwd(), "/Figure_6.jpg"), fixed(" "), ""),
     width=14, height=6, units="in", res=300, pointsize=20)
cor_prod_pred_total
```

```
## `geom_smooth()` using formula 'y ~ x'
```

```
dev.off()
```

```
## pdf
##   2
```

```
cor.test(cor_nor$constr_to_neutral_diff,
         cor_nor$cdi_total,
         method="spearman")
```

```
##
##  Spearman's rank correlation rho
##
## data:  cor_nor$constr_to_neutral_diff and cor_nor$cdi_total
## S = 1032, p-value = 0.3034
## alternative hypothesis: true rho is not equal to 0
## sample estimates:
##        rho
## -0.2647059
```

```r
cor.test(cor_eng$constr_to_neutral_diff,
         cor_eng$cdi_total,
         method="spearman")
```

```
##
##  Spearman's rank correlation rho
##
## data:  cor_eng$constr_to_neutral_diff and cor_eng$cdi_total
## S = 818, p-value = 0.9962
## alternative hypothesis: true rho is not equal to 0
## sample estimates:
##         rho
## -0.00245098
```
